# Factors influencing sedentary behaviour: A system based analysis using Bayesian networks within DEDIPAC

**DOI:** 10.1371/journal.pone.0211546

**Published:** 2019-01-30

**Authors:** Christoph Buck, Anne Loyen, Ronja Foraita, Jelle Van Cauwenberg, Marieke De Craemer, Ciaran Mac Donncha, Jean-Michel Oppert, Johannes Brug, Nanna Lien, Greet Cardon, Iris Pigeot, Sebastien Chastin, on behalf of the DEDIPAC consortium

**Affiliations:** 1 Leibniz Institute for Prevention Research and Epidemiology–BIPS, Bremen, Germany; 2 Department of Public and Occupational Health, Amsterdam Public Health Research Institute, VU University Medical Center, Amsterdam, Netherlands; 3 Research Foundation Flanders (FWO), Brussels, Belgium; 4 Department of Public Health, Ghent University, Ghent, Belgium; 5 Department of Movement and Sports Sciences, Ghent University, Ghent, Belgium; 6 Department of Physical Education and Sport Sciences, University of Limerick, Limerick, Ireland; 7 Department of Nutrition, University Pierre et Marie Curie, Institute of Cardiometabolism And Nutrition (ICAN), Pitie-Salpêtrière Hospital (AP-HP), Paris, France; 8 Amsterdam School for Communication Research, University of Amsterdam, Amsterdam, The Netherlands; 9 Department of Nutrition, University of Oslo, Oslo, Norway; 10 University of Bremen, Faculty of Mathematics and Computer Science, Bremen, Germany; 11 Institute for Applied Health Research, School of Health and Life Science, Glasgow Caledonian University, Glasgow, United Kingdom; Vanderbilt University, UNITED STATES

## Abstract

**Background:**

Decreasing sedentary behaviour (SB) has emerged as a public health priority since prolonged sitting increases the risk of non-communicable diseases. Mostly, the independent association of factors with SB has been investigated, although lifestyle behaviours are conditioned by interdependent factors. Within the DEDIPAC Knowledge Hub, a system of sedentary behaviours (SOS)-framework was created to take interdependency among multiple factors into account. The SOS framework is based on a system approach and was developed by combining evidence synthesis and expert consensus. The present study conducted a Bayesian network analysis to investigate and map the interdependencies between factors associated with SB through the life-course from large scale empirical data.

**Methods:**

Data from the Eurobarometer survey (80.2, 2013) that included the International physical activity questionnaire (IPAQ) short as well as socio-demographic information and questions on perceived environment, health, and psychosocial information were enriched with macro-level data from the Eurostat database. Overall, 33 factors were identified aligned to the SOS-framework to represent six clusters on the individual or regional level: 1) physical health and wellbeing, 2) social and cultural context, 3) built and natural environment, 4) psychology and behaviour, 5) institutional and home settings, 6) policy and economics. A Bayesian network analysis was conducted to investigate conditional associations among all factors and to determine their importance within these networks. Bayesian networks were estimated for the complete (23,865 EU-citizens with complete data) sample and for sex- and four age-specific subgroups. Distance and centrality were calculated to determine importance of factors within each network around SB.

**Results:**

In the young (15–25), adult (26–44), and middle-aged (45–64) groups occupational level was directly associated with SB for both, men and women. Consistently, social class and educational level were indirectly associated within male adult groups, while in women factors of the family context were indirectly associated with SB. Only in older adults, factors of the built environment were relevant with regard to SB, while factors of the home and institutional settings were less important compared to younger age groups.

**Conclusion:**

Factors of the home and institutional settings as well as the social and cultural context were found to be important in the network of associations around SB supporting the priority for future research in these clusters. Particularly, occupational status was found to be the main driver of SB through the life-course. Investigating conditional associations by Bayesian networks gave a better understanding of the complex interplay of factors being associated with SB. This may provide detailed insights in the mechanisms behind the burden of SB to effectively inform policy makers for detailed intervention planning. However, considering the complexity of the issue, there is need for a more comprehensive system of data collection including objective measures of sedentary time.

## Introduction

Sitting has become the dominant posture in most domains of human activity; including work, education, transport, and leisure time, gradually displacing most forms of physical activity over the last fifty years in developed and developing countries [1]. Nowadays people tend to spend a major part of their waking day sitting with 50% of the European population sitting more than 6 hours per day and particularly older adults being sedentary in excess of 60% of their waking day [2, 3]. Activities of daily life that are performed while in a sitting, reclining, or lying posture and that require little energy expenditure are referred to as sedentary behaviour (SB) [4, 5]. High levels of SB are associated with an increased risk of major chronic diseases, loss of independence in later life and premature mortality [6–8].

Consequently, several countries have issued specific recommendations to reduce time spent in sedentary behaviours at all ages as part of their national public health guidelines for physical activity [9, 10]. While the main target is to increase the proportion of time individuals spend in health enhancing moderate-to-vigorous activity, there is evidence that replacing sedentary time with even light intensity physical activity incidental to daily living may confer positive health benefits particularly in the least active segments of the population [11–14].

Understanding the determinants of SB is a necessary step to develop effective interventions and public health policies to curb the rise in sedentary time and reduce its societal burden. Recent reviews on the life-course determinants of SB [15–17] highlighted that we know more about whom such interventions or policies should target than the actual conditions in which individuals and groups sit for a large part of the waking day. In particular, there is a dearth of information about distal determinants and the role of the physical, cultural, social, and policy settings in which people live and work. In addition, it is not clear how determinants at different levels interact.

The present study was undertaken as part of the DEterminants of DIet and Physical Activity (DEDIPAC) Knowledge Hub, which was a joint action as part of the European Joint Programming Initiative “A Healthy Diet for a Healthy Life”[18, 19]. In the scope of DEDIPAC, an international expert consensus and determinant mapping exercise was performed. As a result, the SOS-framework (Systems of Sedentary Behaviours) was developed as an international transdisciplinary consensus framework for the study of determinants, research priorities, and policy on SB across the life-course [20]. The SOS framework is based on a concept mapping approach focusing on understanding the interrelation between factors affecting SB. It was developed through combination of evidence synthesis and expert consensus. Concept mapping is a standardised mixed method, which combines qualitative opinions with multivariate statistical analysis to enable a group to gather and organise ideas into a conceptual framework. As part of the concept mapping approach, factors related to SB were clustered into six different clusters named psychology and behaviour, institutional and home settings (e.g. educational level, social class, or wealth), physical health and wellbeing (e.g. life satisfaction), built and natural environment (e.g. access to green spaces, recreational facilities, transport infrastructures), social and cultural context (e.g. migration background or household size), and politics and economics (e.g. car use or GDP). Eventually, a consensus was built to set priority for future research. Voting on the presented clusters resulted in a 92% consensus that was obtained on a ranking setting factors related to the home and institutional settings to the first priority. From this framework, it was concluded that understanding the complex interplay between determinants is key to identify potential levers for change [19, 20].

Since classical statistical models are not able to reflect complex association structures more recent approaches have to be applied. In this respect, Bayesian network modelling is an appropriate tool that allows to model complex interactions of factors in terms of direct and indirect conditional independencies. Bayesian networks (BNs) represent probabilistic models and are widely used in epidemiology [21], healthcare [22], and intervention planning [23]. In particular, BNs have been successfully used to understand the determinants of obesity [24] and active transport [25] beside the complex causal pathway of diseases [26]. Thus, the aim of this study was to exploit BNs to show how interdependencies between factors particularly associated with SB through the life-course can be comprehensively investigated and mapped based on large scale empirical data.

## Methods

### Study data

The analysis was based on data of the Eurobarometer survey and additional databases. The Eurobarometer is a biannual cross-sectional survey, organised on behalf of the European Commission, covering about 1,000 participants per EU Member State. Sampling and instruments are described elsewhere [27]. For this particular study, data of the Eurobarometer wave 80.2 was used, which was conducted in the 28 EU Member States in 2013. This particular survey included the special Eurobarometer 412 “Sport and physical activity”, including questions on socio-demographic factors, lifestyle behaviours, and the International Physical Activity Questionnaire (IPAQ) short version [28] to assess time spent active and time spent sitting. The European Commission approved the study protocols and informed consent was obtained from all participants. The information was anonymised and de-identified prior to the access to the data.

In addition, data from the Eurostat database (ec.europa.eu/eurostat), the database on Nutrition Obesity and Physical Activity (NOPA, data.euro.who.int/nopa) of the WHO and the European weather database (www.climatedata.eu) were selected to add further information on macro-level variables considering country or within country regions [29, 30].

Overall, 34 variables, including age and sex, were selected aligned to the SOS-framework [20]. Variables are described below per cluster of the SOS-framework. They were operationalised either as binary or as ordered factors to avoid small numbers or missing values within categories in the analyses stratified according to sex and age.

#### Psychology and behaviour

The Eurobarometer assessed *sitting time* on a usual day using the IPAQ short providing ten response categories ranging from 1h or less up to more than 8h 30min. To obtain more balanced samples within each category, sitting time was collapsed into four categories for our analyses, i.e. ‘1h or less up to 2h 30min’ (1), ‘2h 31min up to 4h 30min’ (2), ‘4h 31min up to 7h 30min’ (3), and ‘7h 31min up to 8h 30min or more’ (4).

*Physical activity (PA)* was calculated from reported moderate and vigorous physical activity in the Eurobarometer survey. Information on active days per week and active time per day was collapsed into minutes per week spent in moderate (MPA) or vigorous physical activity (VPA), respectively. PA was categorised as ‘inactive’ (0) for reporting 0 minutes per week of either MPA or VPA per week, ‘low active’ (1) if MPA was lower than 150 minutes **and** VPA was lower than 75 minutes per week, ‘sufficiently active’ (2) for MPA equal or higher than 150 minutes per week **or** VPA equal or higher than 75 minutes per week, and as ‘highly active’ (3) if MPA was equal or higher than 150 minutes per week **and** VPA was equal or higher than 75 minutes per week.

*Life satisfaction* was derived from a four-point Likert scale in the Eurobarometer questioning “On the whole, how satisfied or not are you with the life you lead?” and responses (very, fairly, not very, not at all) were dichotomised as ‘satisfied’ (very or fairly; 1) or ‘not satisfied’ (not very or not at all; 0).

In addition, internet use (*netuse*) was calculated from three questions on internet usage in the Eurobarometer, either at home, at work, or somewhere else. For each of the three locations, six reported categories ranging from ‘everyday’ to ‘about once a week’ were collapsed to three, i.e. ‘everyday’ (2), ‘two or three times per month’ or ‘less often’ (1), and never/no access (0). The highest frequency of one of the three locations was used as the variable *netuse*.

#### Institutional and home settings

*Occupational level* was derived from the reported current occupation (18 response categories) and condensed into eight categories; ‘self-employed’ (1), ‘employed professional or management’ (2), ‘employed position working at desk or travelling’ (3), ‘employed position in service, or skilled or unskilled manual worker’ (4), ‘currently unemployed and responsible for household’ (5), ‘unemployed and not working’ (6), ‘retired or unable to work’ (7), ‘student’ (8).

Categories for *educational level* were built from the age at which the participant reported to have stopped full time education as follows: ‘15 years old or younger’ (1), ‘16 to 19 years old’ (2), ‘20 years old or older’ (3).

Perceived *social class* was derived from three answer categories, i.e. ‘working class’, ‘middle class’, and ‘higher class of society´ based on the question “Do you see yourself and your household belonging to…?” To balance the answer categories, social class was comprised to two categories, i.e. ‘working class’ (0), and ‘middle and higher class’ (1).

*Financial burden* was taken from responses to the question “During the last twelve months, how often have you had difficulties in paying your bills at the end of the month…?”. We dichotomised answer categories as yes (1) for ‘most of the time’, and ‘occasionally’, and no (0) for ‘almost never / never’.

In addition, participants answered yes / no on a list of things they own, e.g. car, television or flat / house, following the question “Which of the following things do you have? “. From this question, we categorised *wealth* as follows: ‘owning a flat / a house which you have finished paying for’ (2), ‘owning a flat / a house which you are still paying for’ (1), ‘none of the above’ (0); ‘owning a computer’ or owning an internet connection (yes / no) were also selected as variables (*computer*, *internet*) from this list and used as factors in the system of institutional and home settings.

From the Eurostat database, the percentage of internet users per country, who were active social media users on a monthly basis, were used for the analysis. We dichotomised the *social media penetration* as above EU average (1) or below (0).

#### Physical health and wellbeing

The Eurobarometer assessed perceived *quality of healthcare* based on a four-point Likert scale, i.e. very good, fairly good, fairly bad, and very bad, asking “How would you evaluate the overall quality of healthcare in your country? “. We dichotomised this scale into ‘good’ (very or fairly good; 1) and ‘bad’ (fairly or very bad; 0).

From the Eurostat database, number of healthcare personnel per 100.000 inhabitants was collected on a regional level (NUTS2) and categorised into tertiles to include the factor *healthcare provision* in our analysis. In addition, country level *prevalence of chronic diseases* was obtained from the same source and categorised as ‘above average’ (1) and ‘below average’ (0).

#### Built and natural environment

Level of *urbanity* was reported by the Eurobarometer participants as living in a ‘rural area or village’ (1), ‘small or medium-sized town’ (2), or living in a ‘large town or city’ (3).

Participants also reported their agreement on a four-point Likert scale on statements about perceived recreational facilities in the neighbourhood, i.e. “The area where you live offers you many opportunities to be physically active“, or “Local sports clubs and other local providers offer many opportunities to be physically active“. Perceived availability of facilities (*facility*) was categorised as ‘high’ (1), if participants agreed (tend to agree or totally agreed) on one of these statements, and ‘low’ (0), if they disagreed (tend to disagree or totally disagreed) to both statements. Moreover, a statement about perceived municipal support (*municipality*), i.e. “Your local authority does not do enough for its citizens in relation to physical activeness“, was dichotomised into ‘high’ (1) or ‘low’ (0) as explained above.

European *region* was categorised according to the geographical regions defined by WHO based on the country in which the participants actually lived. ISO country codes were used to define countries as ‘western or central’ (1), ‘southern’ (2), ‘northern’ (3), or ‘eastern’ (4) regions in Europe.

Data on daily rainfall (*precipitation*) and *temperature* in the year of the survey (2013) was collected from the National and European weather database (e.g. [28]). Maximum annual average temperature was categorised into ‘hot’ (> 15° Celsius), ‘moderate’ (10–15° Celsius) or ‘cold’ (<10° Celsius). In addition, average daily precipitation per day was categorised as ‘dry’ (<0.2mm/day), ‘moderate’ (0.2 – 2mm/day), or ‘wet’ (>2mm/day). Both, temperature and precipitation were aligned to the Eurobarometer using the NUTS2 coding of regions within countries.

#### Social and cultural context

Participants of the Eurobarometer reported on relationship status and number of people within the household. *Household size* was derived by the reported ‘number of children younger than 10 years’, ‘number of children aged 10 to 14’, and ‘number of people aged 15 years or older’ who live within the same household, and was categorised as 0, 1, 2, and 3 or more. Having a *partner* and having *children* was derived as a binary factor from 14 different possible answer categories for the current situation of the relationship, e.g. ‘married (or remarried) and living without children’ (or ‘living with the child of a previous marriage’) or ‘single and living with children’ (or ‘single living without children’) etc.

*Club membership* of health or fitness centres, sports clubs, socio-cultural clubs including sport activities, or other clubs, was categorised as ‘yes’ (1) / ‘no’ (0) for any confirmation of the question “Are you a member of any of the following clubs where you participate in sport or recreational physical activity”.

To identify migration status of EU citizens, we considered the mismatch between the nationality of the participant of the Eurobarometer and the EU-country the survey and categorised as ‘yes’ (1) or ‘no’ (0). Since the Eurobarometer only includes EU-citizens, this factor reflects *EU-migration*. Information from non-EU citizens was not collected by the Eurobarometer.

#### Politics and economics

*Car* ownership (‘yes’ (1)/’no’ (0)) was selected from the list of belongings that Eurobarometer participants reported to own.

On the country level, the availability of policies on SB (*SB guidelines*, ‘yes’ (1) / ‘no’ (0)), physical activity (*PA guidelines*, ‘yes’(1) / ‘no’ (0)) and public transportation (*Transport policy*, ‘yes’ (1) / ‘no’ (0)) was collected from the European databases on Nutrition, Obesity and Physical Activity (NOPA) of the WHO, that provides information on national and subnational policy development. Additionally, the Eurostat database was used to add *GDP* on a regional level (NUTS2 subcountry regions) which was categorised into tertiles.

#### Study sample

The Eurobarometer provided data on 27,919 participants aged 15 and older from 28 European countries. Observations with missing values in any of the 33 variables were excluded, resulting in a total study sample of 23,865 observations. We did not identify any indication for systematic missing mechanisms. Thus, we assumed the data to be missing at random (MAR). However, regional level data (e.g. policy availability or healthcare provision on the NUTS2 subcountry level were not available for Malta (n = 500). Hence, our study was conducted for 27 European countries only. We arbitrarily divided the sample into eight strata based on sex and four age categories, i.e. young (15–25), adults (26–44), middle-aged (45–64), and older adults (65+) in order to account for differences in associations between sexes and over the life-course.

#### Statistical analyses

A Bayesian network (BN) was fitted for the overall study sample as well as for each sex- and age-specific stratum. A BN is a probabilistic model that represents a set of random variables and their conditional independencies via a directed acyclic graph (DAG). A DAG consists of nodes and edges where nodes represent the factors and edges between nodes describe the dependence structure. BNs allow to model conditional dependencies between variables and to capture multicollinearity between variables [31–33].

Since SB and PA are highly collinear [12] we did not consider PA for the final analysis to avoid the BNs being dominated by this well-known relationship and to enable the algorithm to find potential, less studied relationships among the 32 factors instead. However, BNs were re-calculated including PA (considering 33 variables) as sensitivity analysis using the same algorithm.

We applied a hybrid algorithm to identify the topology of a BN that comprises conditional independence tests and network scores [34]. The technical details of the applied methods can be found as supplementary material (see S1 Annex). We used a bootstrap resampling approach to estimate the stability of edges for the entire study sample and each sex- and age-stratum [35]. The complete dataset as well as each of the strata were replicated 1,000 times as part of a random resampling with replacement. For each replication a BN was fitted and for each original dataset (and the strata) replications were condensed to an average network. Stability of any edge was defined as the percentage of the 1,000 BNs in which the edge was found. Edges that were found in at least 40% of the replicated BNs were considered for the average BN. This threshold was chosen to be less conservative in the inclusion of edges in the final average BNs. With regard to the self-reported dataset and the lack of detail in some of the variables, we increased the chance of more false positive associations on purpose in the attempt to provide more insights into possible associations between the considered factors [33]. In addition, results will be only presented as undirected graphs to avoid misinterpretation of edges as causal relationships of factors.

Importance and distance of nodes and their respective clusters were identified by computing network statistics such as network denseness and weighted betweenness centrality (see S1 Annex). Here, weighted betweenness centrality was only calculated for the subgraph of nodes that are linked to SB and weighted considering the bootstrap stability of edges [33]. Distance to SB was used to identify important factors within the BNs and was calculated as the number of edges by which any factor was linked to SB (see S1 Annex). We aggregated this information for each cluster of factors aligned from the SOS-framework in terms of average distance to SB [32].

All analyses were conducted in R using the *bnlearn* package (4.0) [31] in *R* (3.3.0) [36] to derive the BNs. Network statistics were calculated using the *igraph* package (1.01) [37].

## Results

Table 1 presents sample sizes and network statistics for all resulting networks of the whole sample and of all sex- and age-specific strata. The sample was almost sex-balanced with more participants in the age groups from 26 to 44 and 45 to 64 years. Network denseness for the whole study sample was 16.9% between the 32 nodes including age and sex. Network denseness of sex- and age-specific strata considering 30 variables were lower and varied between 6.7% and 12.4% with more associations found in the two middle-age groups than in the youngest and oldest age groups. Study characteristics of the study sample and each stratum, with regard to all 33 variables, are presented as supplementary material in S1 Table. Mean distance of factors within clusters of the SOS-framework is also presented in Table 1, while distance of each factor to SB is presented in detail in S2 Table as supplementary material.

**Table 1:** Sample size and network statistics determining most important nodes by means of weighted betweenness centrality, network denseness and unweighted distance to the factor sedentary behaviour (SB) calculated for the complete sample from the Eurobarometer and for each sex- and age-stratum.

	All	Young female	Young male	Adult female	Adult male	Middle-aged female	Middle-aged male	Older adults female	Older adults male
Sample size: N (%)	23,865	1,218 (5.1)	1,144 (4.8)	3,872 (16.2)	3,244 (13.6)	4,797 (20.1)	3,907 (16.4)	2,612 (10.9)	3,071 (12.9)
**Importance of nodes to SB**									
Highest weighted betweenness centrality	GDP	Occupational level	Occupational Level	Urbanity	Healthcare provision	Urbanity	GDP	Urbanity	Prev. of chronic diseases
2^nd^ highest weighted betweenness centrality	Region	Educational level	Having a partner & Internet use	Healthcare Provision	Occupational level	Car ownership	Urbanity	Facilities	Car ownership
**Network denseness**	16.9%	9.0%	7.1%	12.4%	10.8%	11.3%	12.0%	10.3%	6.7%
**Distance of nodes to SB**^**$**^									
Psychology and behaviour	2.00	3.00	2.00	2.50	2.50	2.50	3.00	3.00	4.00
Institutional and home settings	1.75	2.75	2.60	2.50	2.50	2.75	2.63	4.63	5.29
Physical health and wellbeing	2.33	N.A.	3.00	3.33	4.00	3.33	3.33	3.67	3.33
Built and natural environment	1.67	N.A.	N.A.	2.50	4.17	2.50	2.50	2.83	2.17
Social and cultural context	2.00	2.33	3.00	2.60	3.00	3.50	3.60	6.00	6.50
Politics and economics	2.00	N.A.	N.A.	3.80	3.80	3.60	3.40	5.60	3.20

$: average distance of nodes within a system of the SOS-framework

N.A.: not applicable (no node of this cluster is linked to SB via any edge)

**Fig 1:**
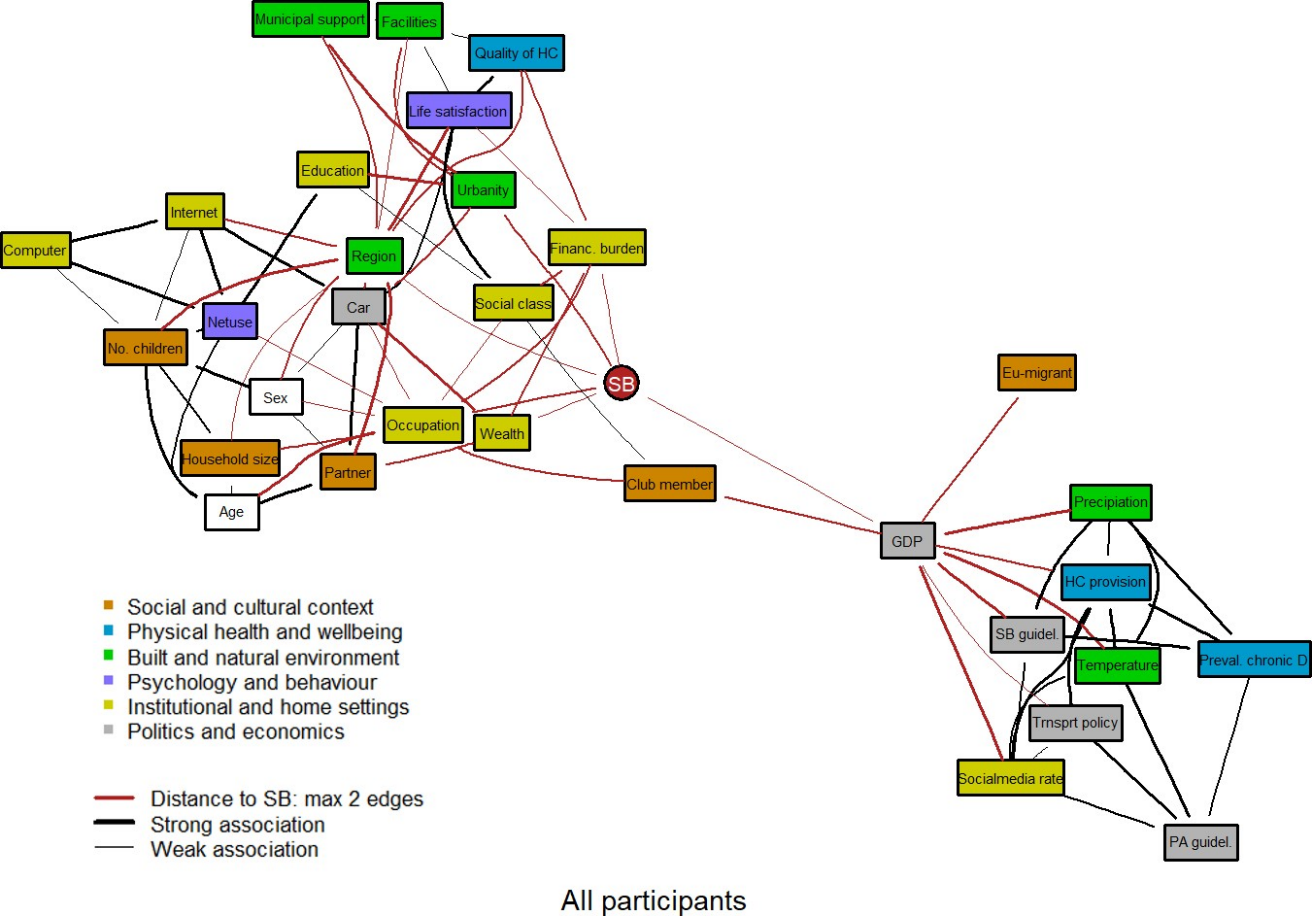
Graph depicting the Bayesian network of 33 factors for the complete study sample (N = 23,865).

The graph of the complete sample is presented in Fig 1. The graph indicates the clusters of factors of the SOS- framework following the attached colour scheme. Edges in red reveal the direct (first) and indirect (second) association of nodes, i.e. factors, with SB. In addition, width of edges indicates the stability of the association as strong (association found in 70% to 100% of bootstrap replications) or weak (association found in 40% to 70% of bootstrap replications).

The graph shows a very complex web of interactions among factors (Fig 1). However, it looks as if the whole BN graph can be decomposed into two parts. Several direct and indirect associations with SB were found via different pathways (see Fig 1). Occupational level, financial burden, wealth, urbanity, European region, and GDP were directly related to SB, while multiple factors of the institutional and home setting as well as the social and cultural context were indirectly linked to SB. Weighted betweenness centrality of nodes revealed that GDP was the most important node supporting most of the network paths followed by region (Table 1). Clusters of institutional and home setting as well as the built and natural environment showed the most direct associations with SB.

**Fig 2:**
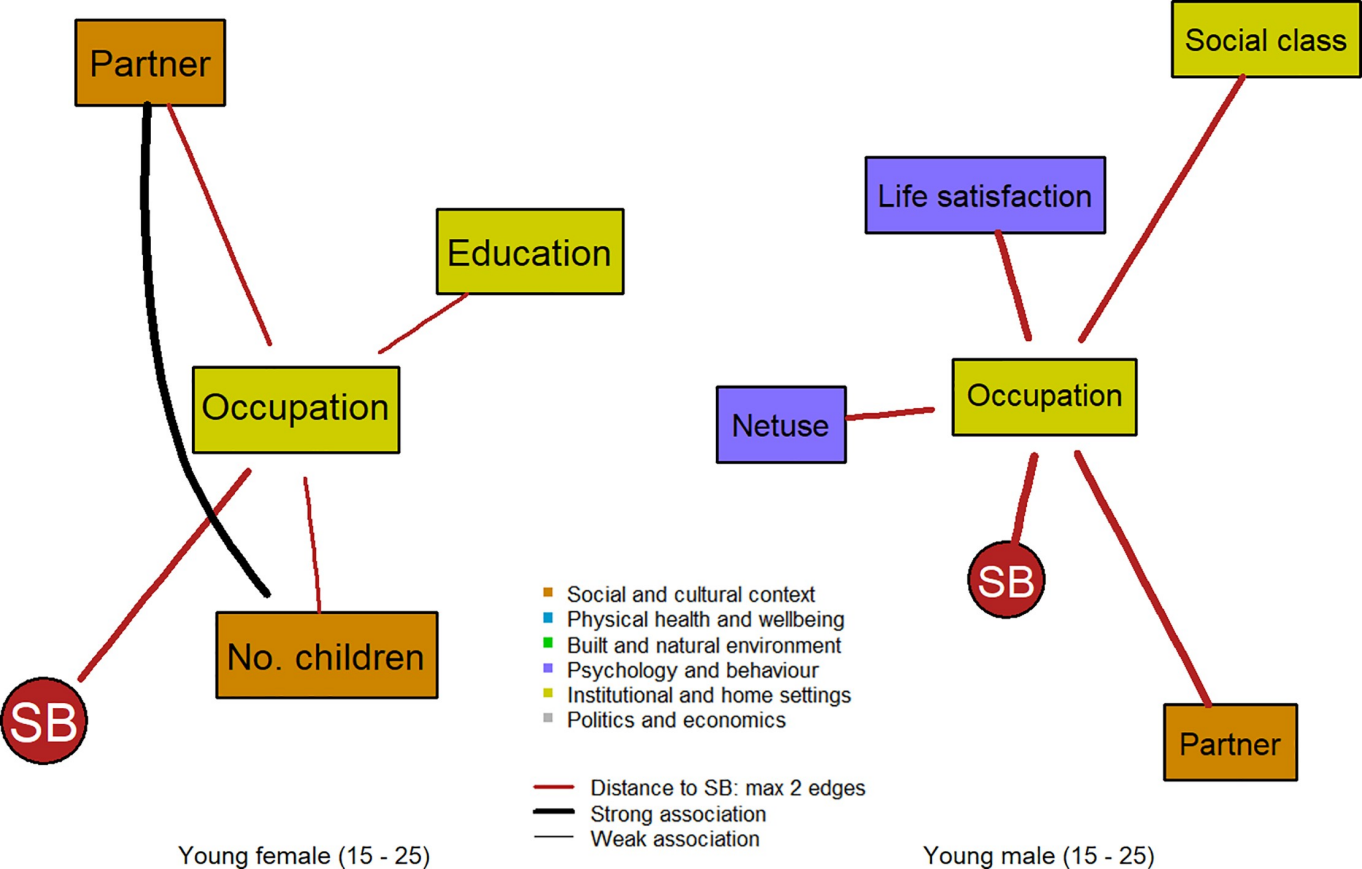
Factors linked to SB within two edges distance in the subgraphs for young females (2a: left; N = 1,218) and young males (2b: right; N = 1,144).

Networks for the various sex- and age-specific strata are provided as supplementary material (see S1 to S8 Figs). To understand the sex differences and changes in their associations among factors over the life-course, subgraphs of the networks for males and females are presented in the manuscript including only nodes that are associated with SB via two edges at most (see Fig 2A and 2B to Fig 5A and 5B). The networks differ in complexity over the life-course depending on age and sex.

**Fig 3:**
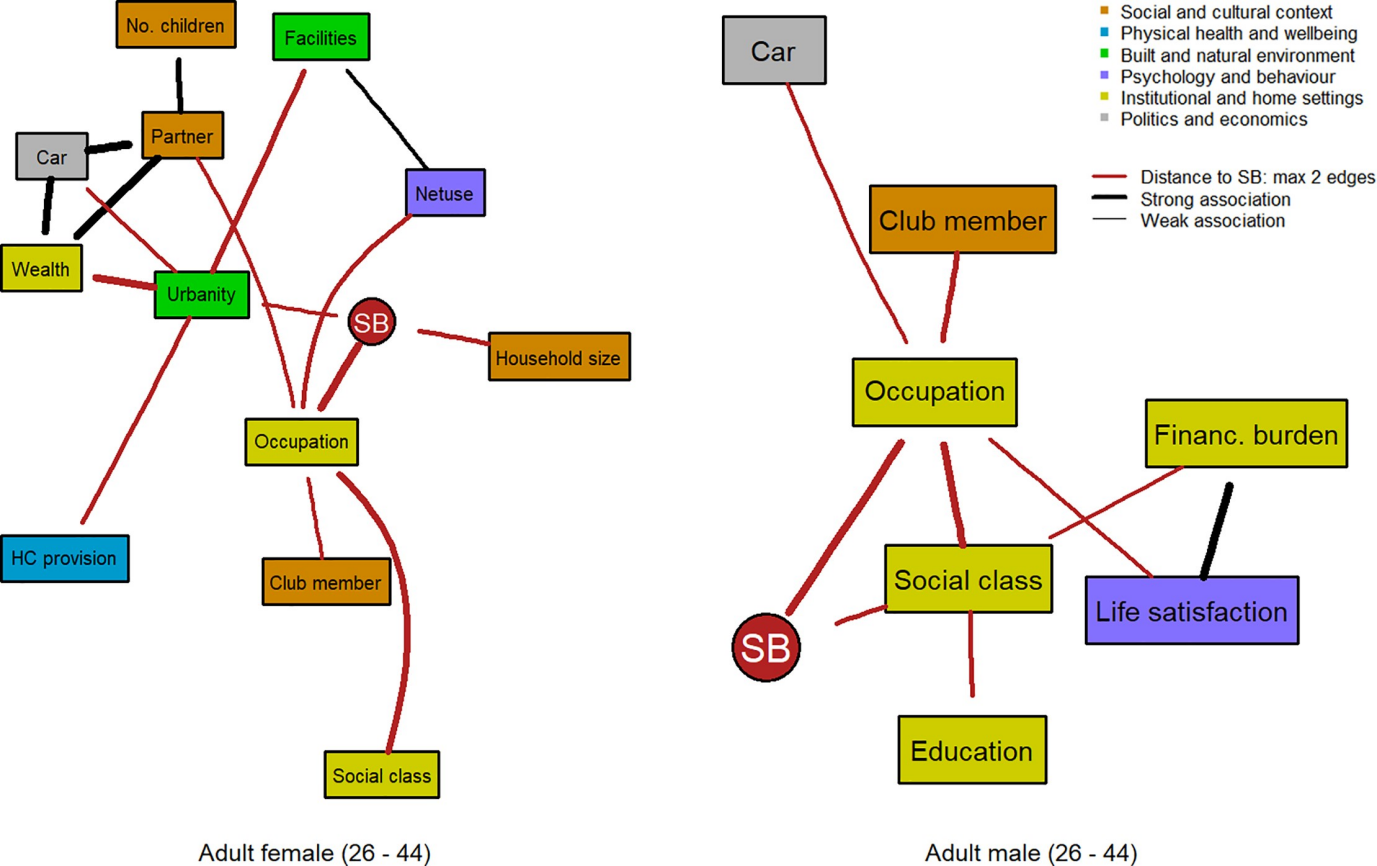
Factors linked to SB within two edges distance in the subgraphs for adult females (3a: left; N = 3,872) and adult males (3b: right; N = 3,244).

**Fig 4:**
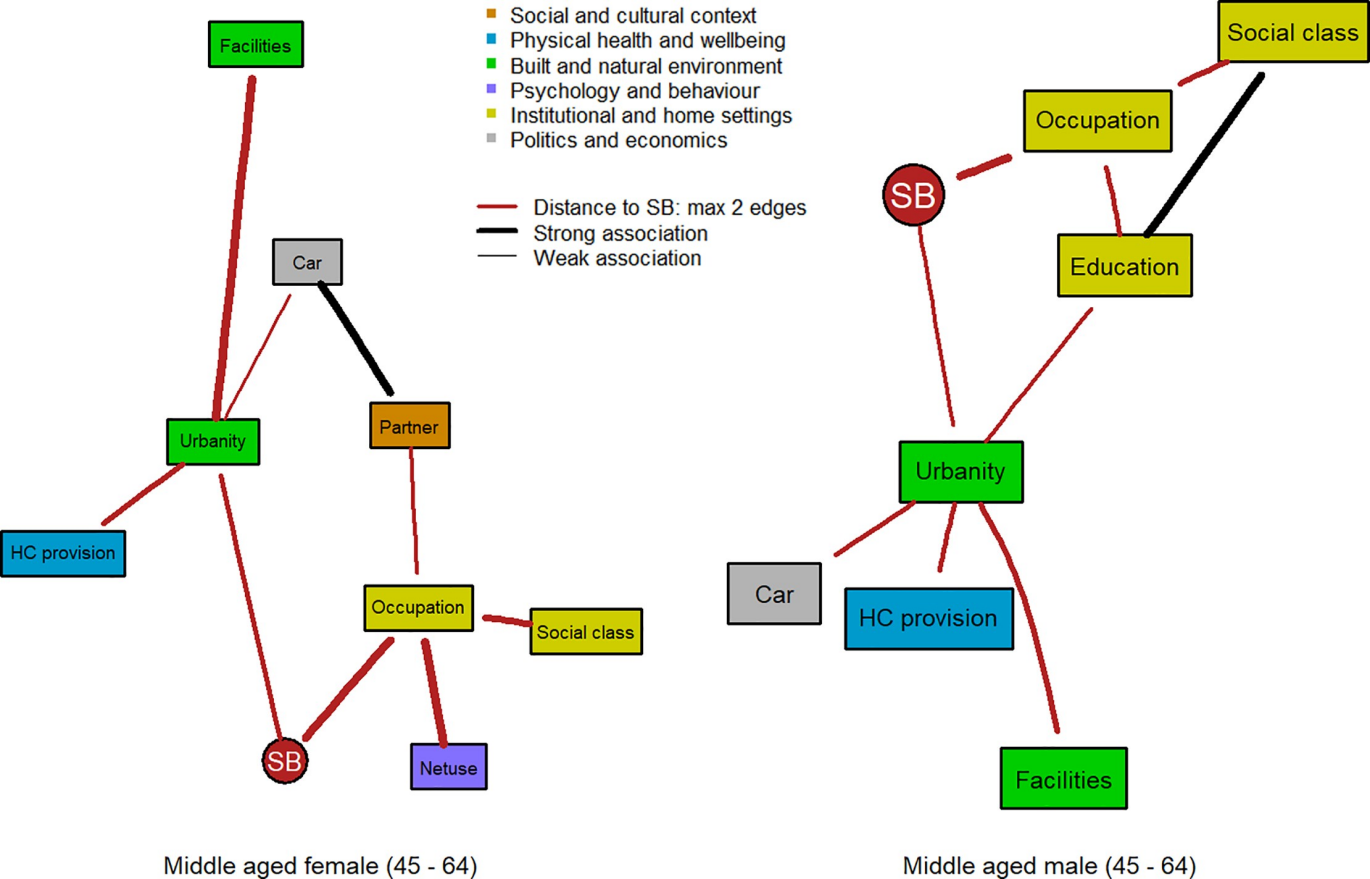
Factors linked to SB within two edges distance in the subgraphs for middle-aged females (4a: left; N = 4,797) and middle-aged males (4b: right; N = 3,907).

**Fig 5:**
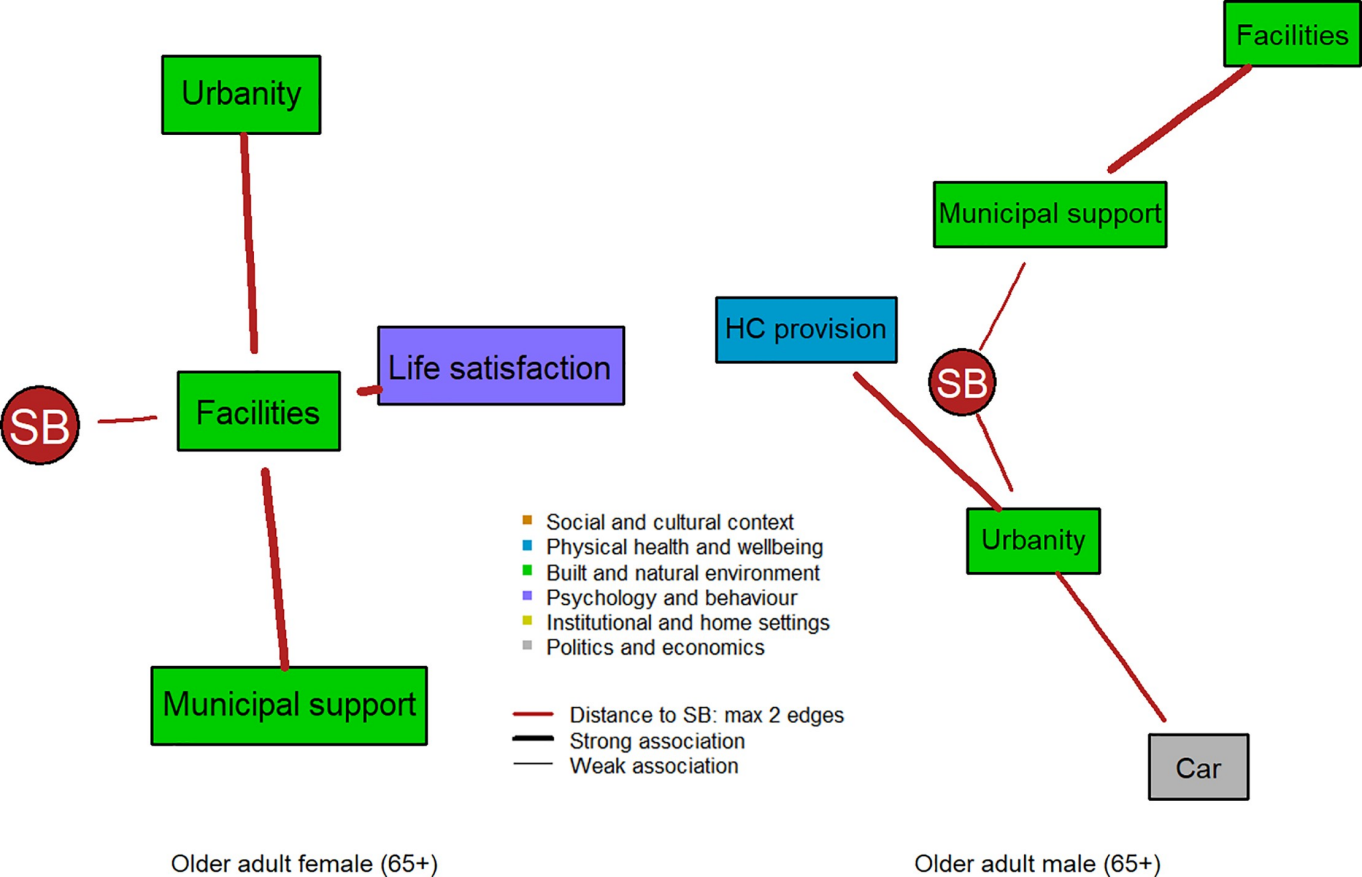
Factors linked to SB within two edges distance in the subgraphs for older adult females (5a: left; N = 2,612) and older adult males (5b: right; 3,071).

SB was linked to occupational level for the first three age groups and for both sexes. In turn, factors of the institutional and home settings, e.g. education, social class, and wealth, as well as factors of the social and cultural context, e.g. number of children and having a partner were indirectly linked to SB, especially via occupational status. Particularly in the youngest two male age groups (Figs 2A and 3A), life satisfaction was consistently found associated with occupational status and indirectly with SB. Household size, having a partner, and number of children were more present in the DAGs for the first three female age groups (Figs 2B, 3B and 4B).

In older adults, SB was only directly associated with factors of the built and natural environment (Fig 5). In older adult females, SB was associated with perceived availability of recreational facilities, which in turn was associated with level of urbanity and perceived support of the municipality, while in older adult males SB was directly associated with perceived municipal support and level of urbanity.

In young adults, for both sexes the most important node was occupational level, while in older age groups urbanity was mostly found as highest or 2^nd^ highest important factor supporting the most paths in the networks for, both, males and females (Table 1).

Shortest mean distance of clusters to SB was found for factors of the institutional and home settings, the social and cultural context and psychology and behaviour with regard to the first three age groups (Table 1). Social and cultural context was consistently closer to SB for females than for males.

In contrast, in older adults, the clusters of institutional and home settings, social and cultural context, and politics and economics showed a very large mean distance to SB compared to networks of younger age groups, while the cluster of the built and natural environment showed the lowest mean distance for, both, males and females (Table 1).

### Sensitivity analyses

Including PA as a factor of the psychological and behavioural system within each BN only slightly changed the graphs in the overall study population as well as in the sex- and age-specific strata (results not shown). However for older males and females, PA was the only factor associated with SB, while club membership was the only factor indirectly associated with SB via PA.

## Discussion

This was the first study which explored and depicted the clustering and interplay between factors that might be associated with SB using BNs. The results showed that, as theoretically expected [20], factors associated with SB build a very complex interacting web with differences between sexes and over the life-course. The network of the overall sample showed a macroscopic structure with two main groups of factors; one group related to individual’s immediate surroundings including factors related to their home and institutional settings and the proximal built environment and the other group related to the broader economic and political context they live in. Interestingly, psychological and behavioural factors that are most often targeted by interventions [38] appeared not to be the factors most closely related to SB. However, only two factors of this cluster were collected by the Eurobarometer survey and could be included in the present analysis.

The graphs showed different patterns throughout the life-course which seemed sex-dependent. Throughout adulthood, factors of the home and institutional settings were directly or indirectly associated with SB, which supports the research priority set by the expert group during the development of the SOS-framework [20]. The graphs of the three adult groups showed a strong association between SB and interrelated factors of the institutional and home settings as well as the social and cultural settings, where the latter have been understudied so far. Occupational level was found to be the main factor associated with SB for all three adult groups before retirement. This is understandable as the type of occupation strongly defines how we spent the majority of our time. Thus, we suggest that interventions that aim to modify the home or institutional settings and particularly the occupational surroundings should take into account multiple factors closely related to the association of occupation and SB, particularly with regard to the cultural and social settings as well.

The fact that the occupational level was directly associated with SB for adult populations is not surprising and this was shown as well in a hierarchical analysis [39]. SB has often been labelled as a “white collar” problem, because white collar workers who mostly live in high GDP regions might engage in more desk-based activities throughout the day. However, this association might be more complex, defined by the relative status of an individual within a region and his/her social and cultural circumstances, especially in young and middle-aged males. Consistently, perceived social class and having a car were indirectly associated with occupational level in the two middle-aged adult groups for both sexes. However, in females, we found a closer link of family-related factors such as having a partner or household size and having children with SB, while for males an association between educational level or social class with SB was consistently present. In this respect, policies and interventions should pay attention to the social and cultural context that has developed around occupational status and social class or the family and that is indirectly linked to SB of the individual.

The influence of the built environment becomes more apparent later in life. In the female adult groups only, we observed a direct link between the level of urbanisation and SB. In older adults, level of urbanisation was the closest factor related to SB in both sexes in line with other factors of the built and natural environment such as the availability of recreational facilities and perceived support from the municipal authorities in promoting active living. The link between urbanisation and SB has been studied cross-sectionally and reported elsewhere [40], where higher population density was found to be associated with more SB for older adults. However, longitudinally, an increase in population density was associated with a reduction in sedentary time at all ages. The global trend towards urbanisation might therefore have a positive impact on reducing SB. However, this will not affect populations equally, since populations in more rural areas do not benefit from this trend [40].

In later life, SB was mainly associated with the availability of recreational places in the environment. This might reflect the ability to engage in social participation via social clubs which take older adults out of their homes where they are mostly sedentary [41, 42]. Factors of the built and natural environment are reported qualitatively by older adults [43] but rarely studied quantitatively [16]. Generally, the results suggest that increasing the provision of outdoor-activities, recreational or not, might be a suitable way to influence SB in older adults.

In the three youngest age groups, clusters of the SOS-framework showed only a small range in distance of edges to SB. Mostly, institutional and home settings had the closest link to SB beside the social and cultural context. However, in older adults, beside the prominent cluster of built and natural environment, other clusters, in particular institutional and home settings and social and cultural context, showed only distal relations to SB. This may be explained by the fact that older adults are less connected to either the occupational setting or the family setting that were more relevant in the middle-aged adult groups [44]. Thus, environmental and municipal support seems to replace the social context they experienced through work or family in earlier years.

Network denseness indicated that the number of associations found among the factors within each sex- and age-group was closely related to the sample size. Lower network denseness was present for the young adult groups which had the smallest sample size as well as for the older adults. Here, the missing links of many factors to SB might be induced by the lack of information in these strata.

Potential important variables that might be capable of dispersing a change in the conditional associations of factors around SB might have been identified in the graphs as most important nodes. For the overall study population, occupational level and GDP were identified as most important nodes. However, a recent study that examined the effect of macroscopic economic factors on SB showed that simply increasing the wealth of an area only benefits a small portion of the populations in terms of reducing SB [40] and warned about potential inequities of solutions disregarding the complex interactions between economic and social factors. Factors of the institutional and home settings as well as economic and environmental factors seem to be important to explain the complex interplay of factors which suggests that acting on those factors might produce changes in SB via different factors that are indirectly associated with SB [45]. Interestingly, potential important factors are not necessarily directly linked to SB, some are even quite distant suggesting that SB might result from profound societal circumstances [1]. Further analyses need to include more detailed information on factors to further identify important variables in BNs that may serve as tipping points for interventions on SB.

### Strengths and limitations

Strength of the study is the use of a large European sample that is assessed by a standardized questionnaire. The use of micro-level and regional-level variables allowed us to cover most of the clusters for the analysis. Eventually, the use of the Bayesian network analysis enabled us to account for the interdependencies between all considered factors. The main limitation of the study is the use of self-reported data which have well-known limited validity and accuracy, particularly with regard to the use of single questions for assessment of factors such as sedentary time. Nevertheless, Eurobarometer provides a large sample that covers the diversity in Europe. Moreover, correlation between IPAQ-based sitting time and accelerometer-based estimated sedentary behaviour indicated moderate agreement [46]. The analysis has also limited variability for macro-level factors at the regional level, which were entered in the network as ordered or binary factors only and might again limit the variability in the models. Thus, close associations among regional-level factors might be seen as a statistical artefact. However, individual level factors were also found to be strongly related to regional level factors. There is a lack of variables and factors included in the analysis. Some clusters of the SOS-framework are underrepresented, particularly the behavioural and psychological cluster, which means that the graphs miss latent information about individual motivations and other unmeasured confounders.

### Implication and use of the networks

The graphs of the BNs provide an empirical validation of the SOS-framework [20]. In this study, the graphs showed a complex interplay of factors around SB and while SB was directly related to only few factors such as occupation and urbanisation these were in turn linked to a complex network of conditional associations with other factors. Based on longitudinal data, the graphs can be useful tools to understand the societal changes that have led to an increase in sedentary time throughout society [1]. Considering cohort data that provide more reliable or preferably objective measures for BN analyses can shed more light on the dynamics of the interrelation of factors around SB and changes in these relationships over the life-course [19]. For example the cohort that was built up by the IDEFICS and I.Family studies [47] incorporated a standardised protocol to collect objective and self-reported data in European children during their transition to adolescence. Such harmonised studies including an extensive survey programme to collect data of objective and reliable measures about behavioural determinants of lifestyle-related diseases need to be promoted on a pan-European level [19].

Building on more complex data, BNs allow for a more detailed interpretation with regard to the graphical assessment of time series, and the assessment of graphs through temporal centrality measures [48]. In addition, the graphs might be suitable to incorporate agent-based computer simulations and scenario-based forecasting [49] to find solutions to decrease sedentary behaviour on a population level by studying the propagation of the potential effect of interventions on different factors. Eventually, BNs provide an extensive analysis of the complexity of factors. Resulting networks can guide interventions and policies to target several factors concurrently while communicating this effectively to policy and decision makers.

## Conclusion

The BN approach provides important insights into the complex interplay of factors related to SB. Aligned to the SOS-framework, this study presents consistent findings of factors related to the home and institutional settings as well as to the social and cultural context clustering around SB and supports the research priorities for these factors. Considering the complexity of the issue, there is need for a more comprehensive system of data collection including objective measures of sedentary time and a multilevel assessment of lifestyle-related factors in these clusters.

### Ethics approval and consent to participate

The European Commission approved study protocols of the Eurobarometer 80.2 (2013) and informed consent was obtained from all participants. The information was anonymised and de-identified prior to the access to the data.

## Supporting information

S1 Annex Technical Annex: Description of theory of Bayesian networks and methods for statistical analyses. (PDF)

S1 Table Study characteristics and number of participants (N/%) per category for each variable included in the Bayesian network analysis. (DOCX)

S2 Table Distance to SB of each factor within corresponding BN. (DOCX)

S1 Fig Graph of young females (16–25 years, N = 1,218). (TIFF)

S2 Fig Graph of young males (16–25 years, N = 1,144). (TIFF)

S3 Fig Graph of adult females (26–44 years, N = 3,872). (TIFF)

S4 Fig Graph of adult males (26–44 years, N = 3,244). (TIFF)

S5 Fig Graph of middle-aged females (45–64 years, N = 4,797). (TIFF)

S6 Fig Graph of middle-aged males (45–64 years, N = 3,907). (TIFF)

S7 Fig Graph of older adult females (65+ years, N = 2,612). (TIFF)

S8 Fig Graph of older adult males (65+ years, N = 3,071). (TIFF)

## Abbreviations

BN: Bayesian network
DEDIPAC: Determinants of diet and physical activity
EU: European Union
IDEFICS: Identification and prevention of Dietary- and lifestyle-induced health EFfects In Children and infantS
IPAQ: International physical activity questionnaire
MPA: Moderate physical activity
MVPA: Moderate to vigorous physical activity
NOPA: database on Nutrition Obesity and Physical Activity
PA: Physical activity
SB: Sedentary behaviour
SOS-framework: Systems of sedentary behaviour framework
WHO: World Health Organization
VPA: Vigorous physical activity
