# Supplementary material for: Factors influencing sedentary behaviour: A system based analysis using Bayesian networks within DEDIPAC
Source: PLoS One. 2019 Jan 30;14(1):e0211546. doi: 10.1371/journal.pone.0211546 (PMC6353197; doi:10.1371/journal.pone.0211546)
Supplement: S1 Annex — (PDF) [file pone.0211546.s001.pdf]

Article: Factors influencing sedentary behaviour: a system based analysis using Bayesian Networks within DEDIPAC

Christoph Buck, Anne Loyen, Ronja Foraita, Jelle Van Cauwenberg, Marieke De Craemer, Ciaran Mac Donncha, Jean-Michel Oppert, Johannes Brug, Nanna Lien, Greet Cardon, Iris Pigeot, Sebastien Chastin, on behalf of the DEDIPAC consortium

Corresponding author
Christoph Buck
Leibniz Institute for Prevention Research and Epidemiology – BIPS, Bremen, Germany.
Tel.: +49 421 218 56944
buck@leibniz-bips.de

Technical Annex

This annex summarises definitions and the technical background of the Bayesian network analysis and the applied network statistics used in this manuscript.

DAG, nodes and edges

A directed acyclic graph (DAG) is a graph (or network) that consists of nodes and edges. Nodes relate to random variables measured in the study which are included in the analysis, and edges indicate the dependence structure of the network in terms of conditional independencies. Two variables are said to be associated with each other when the respective nodes are connected by an edge in the graph, although formally an edge indicates that these two variables are **not** conditionally **in**dependent.

Conditional independence

Conditionally independence is defined as follows: let X, Y, Z be random variables with joint distribution P. We say that X is conditionally independent of Y given Z if and only if

$$P(X = x|Y = y, Z = z) = P(X = x|Z = z).$$

Let us assume that $X =$ sedentary behaviour, $Y =$ occupational level and $Z =$ educational level. Then, the above statement means that “if we know the educational level, then the information on the occupational level does not provide any further information to understand sedentary behaviour”. DAGs represent conditional independencies by a missing edge between two variables.

Bayesian network analysis – Model selection

A Bayesian network is a probabilistic model that represents a set of factors and their conditional dependence structure in terms of a DAG. Bayesian networks combine probability theory and graph theory such that (a) a DAG visualises the structure of a probability model, (b) conditional independencies can directly be read off the DAG and (c) algorithms utilise graph theory for model selection.[1]

Bayesian networks represent a special type of graphical model as a combination of a probabilistic model and graph theory to cope with complex data structures. Graphical models estimate the joint probability distribution of the whole network, instead of applying a set of independent regression analyses, since these are only able to estimate parts of the network, even if they allow for multiple responses. Learning algorithms for Bayesian networks, however, can deal with complex dependence structures and derive an optimal network. Using the concept of conditional independence, the joint probability distribution of all variables in the model can be decomposed into variable-specific conditional probability distributions that only depend on the states of their parent nodes in the DAG. Parents are those nodes from which an arrow originates and is pointing to another node. Any two nodes are conditionally independent given the values of their parents.[2]

Model selection algorithms to estimate the structure of a Bayesian network can be distinguished in (1) constraint-based algorithms, that use conditional independence tests; (2) score-based algorithms, that rank network structures with respect to a goodness-of-fit score; and (3) hybrid algorithms, that combine features of the previous two approaches.[3] All model selection algorithms iteratively select the final network structure. We used the heuristic 2-phase restricted maximisation (RSMAX2) hybrid algorithm which is suitable for mixed discrete and continuous variables under the conditional Gaussian distribution assumption. In the first step the Interleaved Incremental Association algorithm[3] which is based on the Markov blank detection algorithm is used to limit the search space of possible network structures by using Pearson's χ^2 tests for associations between ordinal variables based on an α-level of 0.01 to restrict the number of false positives and to account for the larger sample size. The results of the first steps were used to reduce the remaining number of possible network structures. In the second step the greedy hill-climbing algorithm orients arrows and searches for the optimal network structure, i.e. number of edges, in the restricted space based on the minimised Gaussian log-likelihood. All selection algorithms are suitable for ordinal data in this study and do not take any subject-matter knowledge into account when deciding on the direction of the arrows. Instead, this decision is solely based on the size of the score. Since the aim of the study is mainly exploratory, we considered the skeleton of the BNs, i.e. the graph without any arrows, to avoid misinterpretation of the BNs as causal pathways.[3]

Network statistics

Graph density

The graph density for a network is defined as the frequency of selected edges relative to the number of potential edges, i.e.

$$\mathrm{den(network)} = \frac{\text{\#number of selected edges}}{\binom{\text{\#number of nodes}}{2}}.$$

The graph density is an overall measure for characterising the cohesion of a graph.

Node centrality measures

Centrality measures seek to rank the nodes according to their importance in the network. There are different kinds of centrality measures. Here, the weighted betweenness centrality was applied.

Betweenness centrality

This popular centrality measure is based on the perspective that central nodes of a network are located "between" any other pairs of nodes and are therefore important for the communication flow within a network. A node that lies on many paths between two arbitrary nodes is likely more critical to the information flow. The *betweenness centrality* thus identifies nodes that are located between other pairs of nodes and is calculated as

$$c_B(v) = \sum_{u \neq w \neq v \in V} \frac{\sigma(u, w|v)}{\sigma(u, w)},$$

where V is the set of nodes, $\sigma(u, w|v)$ is the total number of shortest paths between u and w that pass through v, and $\sigma(u, w)$ is the sum over all shortest paths between u and w. In this analysis, we applied the *weighted* betweenness centrality for which the Bootstrap strengths of edges were used as proxy for the strengths of the respective association in the calculation of the shortest paths. Highest and second highest weighted betweenness centralities were used to identify the two most important factors in the BNs around SB.

Literature

1 Bishop C. *Pattern Recognition and Machine Learning*. New York: Springer; 2006.

2 Markowetz F, Spang R. Inferring cellular networks - A review. BMC Bioinf. 2007;8:S5.

3 Scutari M. Learning Bayesian networks with the bnlearn R package. J Stat Softw. 2010;35:1-22.
