# Supplementary material for: Factors influencing sedentary behaviour: A system based analysis using Bayesian networks within DEDIPAC
Source: PLoS One. 2019 Jan 30;14(1):e0211546. doi: 10.1371/journal.pone.0211546 (PMC6353197; doi:10.1371/journal.pone.0211546)
Supplement: S1 Table — (DOCX) [file pone.0211546.s002.docx]

Table S1: Study characteristics and number of participants (N/%) per category for each variable included in the Bayesian Network analysis.

|  |  | *All* | | *Joung (15 - 25 years)* | | | | *Adult(26 - 44 years)* | | | | *Middle aged (45 - 64 years)* | | | | *Older adults (65+ years)* | | | |
| --- | --- | --- | --- | --- | --- | --- | --- | --- | --- | --- | --- | --- | --- | --- | --- | --- | --- | --- | --- |
|  |  |  |  | *female* | | *male* | | *female* | | *male* | | *female* | | *male* | | *female* | | *male* | |
|  |  | *N* | *%* | *N* | *%* | *N* | *%* | *N* | *%* | *N* | *%* | *N* | *%* | *N* | *%* | *N* | *%* | *N* | *%* |
| *Psychology and behaviour* | | | | | | | | | | | | | | | | | | | |
| ***SB*** | | | | | | | | | | | | | | | | | | | |
|  | 1h or less - 2h 30min | 4,100 | 17.2 | 175 | 14.4 | 154 | 13.5 | 877 | 22.6 | 600 | 18.5 | 930 | 19.4 | 678 | 17.4 | 389 | 12.7 | 297 | 11.4 |
|  | 2h 31min - 4h 30min | 6,973 | 29.2 | 323 | 26.5 | 297 | 26.0 | 1,114 | 28.8 | 982 | 30.3 | 1,445 | 30.1 | 1,173 | 30.0 | 899 | 29.3 | 740 | 28.3 |
|  | 4h 31min - 7h 30min | 8,333 | 34.9 | 468 | 38.4 | 445 | 38.9 | 1,187 | 30.7 | 1,003 | 30.9 | 1,618 | 33.7 | 1,302 | 33.3 | 1,252 | 40.8 | 1,058 | 40.5 |
|  | 7h 31min or more | 4,459 | 18.7 | 252 | 20.7 | 248 | 21.7 | 694 | 17.9 | 659 | 20.3 | 804 | 16.8 | 754 | 19.3 | 531 | 17.3 | 517 | 19.8 |
| ***PA*** | | | | | | | | | | | | | | | | | | | |
|  | inactive | 13,019 | 54.6 | 605 | 49.7 | 355 | 31.0 | 2,186 | 56.5 | 1,304 | 40.2 | 2,844 | 59.3 | 1,937 | 49.6 | 2,149 | 70.0 | 1,639 | 62.7 |
|  | low active | 1,132 | 4.7 | 65 | 5.3 | 49 | 4.3 | 206 | 5.3 | 116 | 3.6 | 227 | 4.7 | 175 | 4.5 | 171 | 5.6 | 123 | 4.7 |
|  | sufficiently active | 2,223 | 9.3 | 162 | 13.3 | 148 | 13.0 | 405 | 10.5 | 398 | 12.2 | 418 | 8.8 | 351 | 9.0 | 157 | 5.2 | 184 | 7.1 |
|  | highly active | 7,491 | 31.4 | 386 | 31.7 | 592 | 51.7 | 1,075 | 27.8 | 1,426 | 44.0 | 1,308 | 27.3 | 1,444 | 37.0 | 594 | 19.3 | 666 | 25.5 |
| ***Life satisfaction*** | | | | | | | | | | | | | | | | | | | |
|  | not satisfied | 5,123 | 21.5 | 197 | 16.2 | 210 | 18.4 | 824 | 21.3 | 701 | 21.6 | 1,219 | 25.4 | 944 | 24.2 | 609 | 19.8 | 419 | 16.0 |
|  | satisfied | 18,742 | 78.5 | 1,021 | 83.8 | 934 | 81.6 | 3,048 | 78.7 | 2,543 | 78.4 | 3,578 | 74.6 | 2,963 | 75.8 | 2,462 | 80.2 | 2,193 | 84.0 |
| ***Internet use*** | | | | | | | | | | | | | | | | | | | |
|  | never / no access | 2,938 | 12.3 | 24 | 2.0 | 25 | 2.2 | 146 | 3.8 | 112 | 3.5 | 606 | 12.6 | 429 | 11.0 | 950 | 30.9 | 646 | 24.7 |
|  | two or three time a month or less | 4,252 | 17.8 | 31 | 2.5 | 24 | 2.1 | 272 | 7.0 | 183 | 5.6 | 1,008 | 21.0 | 714 | 18.3 | 1,196 | 38.9 | 824 | 31.5 |
|  | about once a week - everyday | 16,675 | 69.9 | 1,163 | 95.5 | 1,095 | 95.7 | 3,454 | 89.2 | 2,949 | 90.9 | 3,183 | 66.4 | 2,764 | 70.7 | 925 | 30.1 | 1,142 | 43.7 |
|  | | | | | | | | | | | | | | | | | | | |
| *Institutional and home settings* | | | | | | | | | | | | | | | | | | | |
| ***Occupational level*** | | | | | | | | | | | | | | | | | | | |
|  | Self-employed | 1,767 | 7.4 | 21 | 1.7 | 29 | 2.5 | 267 | 6.9 | 425 | 13.1 | 348 | 7.3 | 546 | 14.0 | 38 | 1.2 | 93 | 3.6 |
|  | employed professional or management | 2,413 | 10.1 | 45 | 3.7 | 49 | 4.3 | 621 | 16.0 | 577 | 17.8 | 538 | 11.2 | 521 | 13.3 | 25 | 0.8 | 37 | 1.4 |
|  | employed position at desk / travelling | 2,871 | 12.0 | 125 | 10.3 | 82 | 7.2 | 843 | 21.8 | 597 | 18.4 | 723 | 15.1 | 440 | 11.3 | 35 | 1.1 | 26 | 1.0 |
|  | employed position in service / (un)skilled | 4,839 | 20.3 | 180 | 14.8 | 234 | 20.5 | 989 | 25.5 | 1,179 | 36.3 | 1,113 | 23.2 | 1,034 | 26.5 | 54 | 1.8 | 56 | 2.1 |
|  | responsible for household | 1,549 | 6.5 | 102 | 8.4 | 10 | 0.9 | 541 | 14.0 | 26 | 0.8 | 588 | 12.3 | 30 | 0.8 | 234 | 7.6 | 18 | 0.7 |
|  | unemployed and not working | 1,896 | 7.9 | 176 | 14.4 | 143 | 12.5 | 486 | 12.6 | 330 | 10.2 | 350 | 7.3 | 390 | 10.0 | 13 | 0.4 | 8 | 0.3 |
|  | retired or unable to work | 7,231 | 30.3 | 2 | 0.2 | 4 | 0.3 | 50 | 1.3 | 59 | 1.8 | 1,133 | 23.6 | 941 | 24.1 | 2,671 | 87.0 | 2,371 | 90.8 |
|  | student | 1,299 | 5.4 | 567 | 46.6 | 593 | 51.8 | 75 | 1.9 | 51 | 1.6 | 4 | 0.1 | 5 | 0.1 | 1 | 0.0 | 3 | 0.1 |
| ***Educational level*** | | | | | | | | | | | | | | | | | | | |
|  | 15 years or less | 4,182 | 17.5 | 45 | 3.7 | 45 | 3.9 | 280 | 7.2 | 240 | 7.4 | 841 | 17.5 | 599 | 15.3 | 1,234 | 40.2 | 898 | 34.4 |
|  | 16 to 19 years | 11,899 | 49.9 | 962 | 79.0 | 921 | 80.5 | 1,881 | 48.6 | 1,686 | 52.0 | 2,404 | 50.1 | 2,031 | 52.0 | 1,120 | 36.5 | 894 | 34.2 |
|  | 20 years or more | 7,784 | 32.6 | 211 | 17.3 | 178 | 15.6 | 1,711 | 44.2 | 1,318 | 40.6 | 1,552 | 32.4 | 1,277 | 32.7 | 717 | 23.3 | 820 | 31.4 |
| ***Social class*** | | | | | | | | | | | | | | | | | | | |
|  | working class | 11,121 | 46.6 | 504 | 41.4 | 483 | 42.2 | 1,652 | 42.7 | 1,507 | 46.5 | 2,270 | 47.3 | 1,940 | 49.7 | 1,546 | 50.3 | 1,219 | 46.7 |
|  | middle and higher class | 12,744 | 53.4 | 714 | 58.6 | 661 | 57.8 | 2,220 | 57.3 | 1,737 | 53.6 | 2,527 | 52.7 | 1,967 | 50.4 | 1,525 | 49.7 | 1,393 | 53.4 |
| ***Financial burden*** | | | | | | | | | | | | | | | | | | | |
|  | Yes | 9,624 | 40.3 | 582 | 47.8 | 518 | 45.3 | 1,979 | 51.1 | 1,482 | 45.7 | 2,016 | 42.0 | 1,517 | 38.8 | 918 | 29.9 | 612 | 23.4 |
|  | No | 14,241 | 59.7 | 636 | 52.2 | 626 | 54.7 | 1,893 | 48.9 | 1,762 | 54.3 | 2,781 | 58.0 | 2,390 | 61.2 | 2,153 | 70.1 | 2,000 | 76.6 |
| ***Wealth*** | | | | | | | | | | | | | | | | | | | |
|  | none | 5,658 | 23.7 | 538 | 44.2 | 468 | 40.9 | 1,013 | 26.2 | 932 | 28.7 | 925 | 19.3 | 735 | 18.8 | 630 | 20.5 | 417 | 16.0 |
|  | own house/flat and still paying for | 6,212 | 26.0 | 298 | 24.5 | 217 | 19.0 | 1,603 | 41.4 | 1,236 | 38.1 | 1,108 | 23.1 | 1,023 | 26.2 | 349 | 11.4 | 378 | 14.5 |
|  | own house/flat | 11,995 | 50.3 | 382 | 31.4 | 459 | 40.1 | 1,256 | 32.4 | 1,076 | 33.2 | 2,764 | 57.6 | 2,149 | 55.0 | 2,092 | 68.1 | 1,817 | 69.6 |
| ***Owning a computer*** | | | | | | | | | | | | | | | | | | | |
|  | No | 5,892 | 24.7 | 95 | 7.8 | 78 | 6.8 | 357 | 9.2 | 328 | 10.1 | 1,127 | 23.5 | 861 | 22.0 | 1,829 | 59.6 | 1,217 | 46.6 |
|  | Yes | 17,973 | 75.3 | 1,123 | 92.2 | 1,066 | 93.2 | 3,515 | 90.8 | 2,916 | 89.9 | 3,670 | 76.5 | 3,046 | 78.0 | 1,242 | 40.4 | 1,395 | 53.4 |
| ***Owning internet connection*** | | | | | | | | | | | | | | | | | | | |
|  | No | 6,328 | 26.5 | 125 | 10.3 | 106 | 9.3 | 423 | 10.9 | 389 | 12.0 | 1,205 | 25.1 | 924 | 23.6 | 1,888 | 61.5 | 1,268 | 48.5 |
|  | Yes | 17,537 | 73.5 | 1,093 | 89.7 | 1,038 | 90.7 | 3,449 | 89.1 | 2,855 | 88.0 | 3,592 | 74.9 | 2,983 | 76.4 | 1,183 | 38.5 | 1,344 | 51.5 |
| ***Socialmedia penetration*** | | | | | | | | | | | | | | | | | | | |
|  | below EU average | 12,298 | 51.5 | 665 | 54.6 | 632 | 55.2 | 2,061 | 53.2 | 1,736 | 53.5 | 2,533 | 52.8 | 2,071 | 53.0 | 1,422 | 46.3 | 1,178 | 45.1 |
|  | above EU average | 11,567 | 48.5 | 553 | 45.4 | 512 | 44.8 | 1,811 | 46.8 | 1,508 | 46.5 | 2,264 | 47.2 | 1,836 | 47.0 | 1,649 | 53.7 | 1,434 | 54.9 |
|  | | | | | | | | | | | | | | | | | | | |
| *Physical health and wellbeing* | | | | | | | | | | | | | | | | | | | |
| ***Quality of Healthcare*** | | | | | | | | | | | | | | | | | | | |
|  | bad | 7,188 | 30.1 | 406 | 33.3 | 390 | 34.1 | 1,356 | 35.0 | 1,045 | 32.2 | 1,566 | 32.6 | 1,114 | 28.5 | 742 | 24.2 | 569 | 21.8 |
|  | good | 16,677 | 69.9 | 812 | 66.7 | 754 | 65.9 | 2,516 | 65.0 | 2,199 | 67.8 | 3,231 | 67.4 | 2,793 | 71.5 | 2,329 | 75.8 | 2,043 | 78.2 |
| ***Healthcare provision (personnel per 100.000 hab.)*** | | | | | | | | | | | | | | | | | | | |
|  | lowest tertile | 5,254 | 22.0 | 272 | 22.3 | 217 | 19.0 | 883 | 22.8 | 710 | 21.9 | 1,179 | 24.6 | 867 | 22.2 | 640 | 20.8 | 486 | 18.6 |
|  | middle tertile | 11,184 | 46.9 | 609 | 50.0 | 541 | 47.3 | 1,780 | 46.0 | 1,502 | 46.3 | 2,180 | 45.4 | 1,840 | 47.1 | 1,489 | 48.5 | 1,243 | 47.6 |
|  | highest tertile | 7,427 | 31.1 | 337 | 27.7 | 386 | 33.7 | 1,209 | 31.2 | 1,032 | 31.8 | 1,438 | 30.0 | 1,200 | 30.7 | 942 | 30.7 | 883 | 33.8 |
| ***Prevalence of CVD*** | | | | | | | | | | | | | | | | | | | |
|  | below average | 13,529 | 56.7 | 718 | 58.9 | 651 | 56.9 | 2,314 | 59.8 | 1,935 | 59.7 | 2,688 | 56.0 | 2,212 | 56.7 | 1,611 | 52.5 | 1,400 | 53.6 |
|  | above average | 10,336 | 43.3 | 500 | 41.1 | 493 | 43.1 | 1,558 | 40.2 | 1,309 | 40.4 | 2,109 | 44.0 | 1,695 | 43.4 | 1,460 | 47.5 | 1,212 | 46.4 |
|  | | | | | | | | | | | | | | | | | | | |
| *Built and natural environment* | | | | | | | | | | | | | | | | | | | |
| ***Urbanity*** | | | | | | | | | | | | | | | | | | | |
|  | rural area or village | 8,082 | 33.9 | 365 | 30.0 | 327 | 28.6 | 1,255 | 32.4 | 1,052 | 32.4 | 1,678 | 35.0 | 1,430 | 36.6 | 1,031 | 33.6 | 944 | 36.1 |
|  | small or medium sized town | 8,984 | 37.6 | 456 | 37.4 | 427 | 37.3 | 1,449 | 37.4 | 1,192 | 36.7 | 1,802 | 37.6 | 1,470 | 37.6 | 1,208 | 39.3 | 980 | 37.5 |
|  | large town or city | 6,799 | 28.5 | 397 | 32.6 | 390 | 34.1 | 1,168 | 30.2 | 1,000 | 30.8 | 1,317 | 27.5 | 1,007 | 25.8 | 832 | 27.1 | 688 | 26.3 |
| ***Availability of recreational facilities*** | | | | | | | | | | | | | | | | | | | |
|  | high | 4,912 | 20.5 | 235 | 19.3 | 188 | 16.4 | 829 | 21.4 | 613 | 18.9 | 1,039 | 21.7 | 781 | 19.9 | 681 | 22.1 | 546 | 21.0 |
|  | low | 18,953 | 79.4 | 983 | 80.7 | 956 | 83.6 | 3,043 | 78.6 | 2,631 | 81.1 | 3,758 | 78.3 | 3,126 | 80.0 | 2,390 | 77.8 | 2,066 | 79.1 |
| ***Municipal support*** | | | | | | | | | | | | | | | | | | | |
|  | high | 14,577 | 61.1 | 645 | 53.0 | 664 | 58.1 | 2,243 | 57.9 | 1,809 | 55.7 | 2,961 | 61.8 | 2,390 | 61.1 | 2,142 | 69.7 | 1,723 | 66.0 |
|  | low | 9,288 | 38.9 | 573 | 47.0 | 480 | 42.0 | 1,629 | 42.1 | 1,435 | 44.2 | 1,836 | 38.3 | 1,517 | 38.8 | 929 | 30.3 | 889 | 34.0 |
| ***European Region (ISO Code according to WHO)*** | | | | | | | | | | | | | | | | | | | |
|  | western | 8,492 | 35.6 | 494 | 40.6 | 463 | 40.5 | 1,511 | 39.0 | 1,252 | 38.6 | 1,845 | 38.5 | 1,388 | 35.5 | 931 | 30.3 | 608 | 23.3 |
|  | southern | 5,609 | 23.5 | 252 | 20.7 | 196 | 17.1 | 795 | 20.5 | 653 | 20.1 | 997 | 20.8 | 882 | 22.6 | 953 | 31.0 | 881 | 33.7 |
|  | northern | 5,607 | 23.5 | 259 | 21.3 | 264 | 23.1 | 811 | 20.9 | 697 | 21.5 | 1,129 | 23.5 | 1,018 | 26.1 | 751 | 24.5 | 678 | 26.0 |
|  | eastern | 4,157 | 17.4 | 213 | 17.5 | 221 | 19.3 | 755 | 19.5 | 642 | 19.8 | 826 | 17.2 | 619 | 15.8 | 436 | 14.2 | 445 | 17.0 |
| ***Precipitation (avg./year)*** | | | | | | | | | | | | | | | | | | | |
|  | dry (< 0.2mm/day) | 8,398 | 35.2 | 389 | 31.9 | 413 | 36.1 | 1,377 | 35.6 | 1,199 | 37.0 | 1,723 | 35.9 | 1,391 | 35.6 | 999 | 32.5 | 907 | 34.7 |
|  | normal (0.2 mm/day - 2 mm/day) | 12,676 | 53.1 | 689 | 56.6 | 614 | 53.7 | 2,004 | 51.8 | 1,703 | 52.5 | 2,570 | 53.6 | 2,117 | 54.2 | 1,651 | 53.8 | 1,328 | 50.8 |
|  | wet (> 2 mm/day) | 2,791 | 11.7 | 140 | 11.5 | 117 | 10.2 | 491 | 12.7 | 342 | 10.5 | 504 | 10.5 | 399 | 10.2 | 421 | 13.7 | 377 | 14.4 |
| ***Temperature (avg./year)*** | | | | | | | | | | | | | | | | | | | |
|  | cold (< 10°C) | 4,653 | 19.5 | 232 | 19.0 | 206 | 18.0 | 613 | 15.8 | 533 | 16.4 | 909 | 18.9 | 770 | 19.7 | 761 | 24.8 | 629 | 24.1 |
|  | moderate (10°C - 15°C) | 17,785 | 74.5 | 893 | 73.3 | 872 | 76.2 | 3,008 | 77.7 | 2,485 | 76.6 | 3,596 | 75.0 | 2,927 | 74.9 | 2,161 | 70.4 | 1,843 | 70.6 |
|  | hot (> 15°C) | 1,427 | 6.0 | 93 | 7.6 | 66 | 5.8 | 251 | 6.5 | 226 | 7.0 | 292 | 6.1 | 210 | 5.4 | 149 | 4.9 | 140 | 5.4 |
|  | | | | | | | | | | | | | | | | | | | |
| *Social and cultural context* | | | | | | | | | | | | | | | | | | | |
| ***Houshold size (# of.people that live together)*** | | | | | | | | | | | | | | | | | | | |
|  | zero | 17,755 | 74.4 | 844 | 69.3 | 902 | 78.8 | 1,299 | 33.5 | 1,638 | 50.5 | 4,172 | 87.0 | 3,313 | 84.8 | 3,009 | 98.0 | 2,578 | 98.7 |
|  | one | 3,145 | 13.2 | 260 | 21.3 | 179 | 15.6 | 1,143 | 29.5 | 731 | 22.5 | 423 | 8.8 | 359 | 9.2 | 33 | 1.1 | 17 | 0.7 |
|  | two | 2,236 | 9.4 | 78 | 6.4 | 42 | 3.7 | 1,072 | 27.7 | 652 | 20.1 | 161 | 3.4 | 192 | 4.9 | 25 | 0.8 | 14 | 0.5 |
|  | three or more | 729 | 3.1 | 36 | 3.0 | 21 | 1.8 | 358 | 9.2 | 223 | 6.9 | 41 | 0.9 | 43 | 1.1 | 4 | 0.1 | 3 | 0.1 |
| ***Having a partner*** | | | | | | | | | | | | | | | | | | | |
|  | single | 8,347 | 35.0 | 721 | 59.2 | 831 | 72.6 | 901 | 23.3 | 914 | 28.2 | 1,554 | 32.4 | 938 | 24.0 | 1,766 | 57.5 | 722 | 27.6 |
|  | living with partner | 15,518 | 65.0 | 497 | 40.8 | 313 | 27.4 | 2,971 | 76.7 | 2,330 | 71.8 | 3,243 | 67.6 | 2,969 | 76.0 | 1,305 | 42.5 | 1,890 | 72.4 |
| ***Having children*** | | | | | | | | | | | | | | | | | | | |
|  | no | 14,769 | 61.9 | 954 | 78.3 | 1,035 | 90.5 | 1,039 | 26.8 | 1,546 | 47.7 | 2,706 | 56.4 | 2,363 | 60.5 | 2,762 | 89.9 | 2,364 | 90.5 |
|  | living with children | 9,096 | 38.1 | 264 | 21.7 | 109 | 9.5 | 2,833 | 73.2 | 1,698 | 52.3 | 2,091 | 43.6 | 1,544 | 39.5 | 309 | 10.1 | 248 | 9.5 |
| ***Member of a sports or recreational club*** | | | | | | | | | | | | | | | | | | | |
|  | no | 18,464 | 77.4 | 837 | 68.7 | 617 | 53.9 | 2,955 | 76.3 | 2,288 | 70.5 | 3,919 | 81.7 | 3,100 | 79.3 | 2,644 | 86.1 | 2,104 | 80.6 |
|  | yes | 5,401 | 22.6 | 381 | 31.3 | 527 | 46.1 | 917 | 23.7 | 956 | 29.5 | 878 | 18.3 | 807 | 20.7 | 427 | 13.9 | 508 | 19.4 |
| ***EU migrant (EU citizen living in a different EU country)*** | | | | | | | | | | | | | | | | | | | |
|  | no | 23,281 | 97.6 | 1,174 | 96.4 | 1,117 | 97.6 | 3,725 | 96.2 | 3,121 | 96.2 | 4,714 | 98.3 | 3,831 | 98.1 | 3,022 | 98.4 | 2,577 | 98.7 |
|  | yes | 584 | 2.4 | 44 | 3.6 | 27 | 2.4 | 147 | 3.8 | 123 | 3.8 | 83 | 1.7 | 76 | 1.9 | 49 | 1.6 | 35 | 1.3 |
|  | | | | | | | | | | | | | | | | | | | |
| *Politics and economics* | | | | | | | | | | | | | | | | | | | |
| ***Owning a car*** | | | | | | | | | | | | | | | | | | | |
|  | no | 6,579 | 27.6 | 452 | 37.1 | 383 | 33.5 | 793 | 20.5 | 632 | 19.5 | 1,235 | 25.7 | 788 | 20.2 | 1,571 | 51.2 | 725 | 27.8 |
|  | yes | 17,286 | 72.4 | 766 | 62.9 | 761 | 66.5 | 3,079 | 79.5 | 2,612 | 80.5 | 3,562 | 74.3 | 3,119 | 79.8 | 1,500 | 48.8 | 1,887 | 72.2 |
| ***Country-level guidelines to reduce SB*** | | | | | | | | | | | | | | | | | | | |
|  | no | 20,858 | 87.4 | 1,046 | 85.9 | 981 | 85.8 | 3,426 | 88.5 | 2,875 | 88.6 | 4,282 | 89.3 | 3,470 | 88.8 | 2,554 | 83.2 | 2,224 | 85.1 |
|  | yes | 3,007 | 12.6 | 172 | 14.1 | 163 | 14.2 | 446 | 11.5 | 369 | 11.4 | 515 | 10.7 | 437 | 11.2 | 517 | 16.8 | 388 | 14.9 |
| ***Country-level guidelines for PA promotion*** | | | | | | | | | | | | | | | | | | | |
|  | no | 7,826 | 32.8 | 418 | 34.3 | 372 | 32.5 | 1,392 | 36.0 | 1,148 | 35.4 | 1,703 | 35.5 | 1,263 | 32.3 | 842 | 27.4 | 688 | 26.3 |
|  | yes | 16,039 | 67.2 | 800 | 65.7 | 772 | 67.5 | 2,480 | 64.0 | 2,096 | 64.6 | 3,094 | 64.5 | 2,644 | 67.7 | 2,229 | 72.6 | 1,924 | 73.7 |
| ***Country-level policy for public transport*** | | | | | | | | | | | | | | | | | | | |
|  | no | 7,266 | 30.4 | 431 | 35.4 | 405 | 35.4 | 1,249 | 32.3 | 1,089 | 33.6 | 1,511 | 31.5 | 1,126 | 28.8 | 804 | 26.2 | 651 | 24.9 |
|  | yes | 16,599 | 69.6 | 787 | 64.6 | 739 | 64.6 | 2,623 | 67.7 | 2,155 | 66.4 | 3,286 | 68.5 | 2,781 | 71.2 | 2,267 | 73.8 | 1,961 | 75.1 |
| **GDP (regional-level)** | | | | | | | | | | | | | | | | | | | |
|  | lowest tertile | 11,040 | 46.3 | 629 | 51.6 | 612 | 53.5 | 1,979 | 51.1 | 1,592 | 49.1 | 2,360 | 49.2 | 1,744 | 44.6 | 1,225 | 39.9 | 899 | 34.4 |
|  | middle tertile | 6,592 | 27.6 | 305 | 25.0 | 282 | 24.7 | 985 | 25.4 | 898 | 27.7 | 1,236 | 25.8 | 1,117 | 28.6 | 943 | 30.7 | 826 | 31.6 |
|  | highest tertile | 6,233 | 26.1 | 284 | 23.3 | 250 | 21.9 | 908 | 23.5 | 754 | 23.2 | 1,201 | 25.0 | 1,046 | 26.8 | 903 | 29.4 | 887 | 34.0 |
