# Supplementary material for: Factors influencing sedentary behaviour: A system based analysis using Bayesian networks within DEDIPAC
Source: PLoS One. 2019 Jan 30;14(1):e0211546. doi: 10.1371/journal.pone.0211546 (PMC6353197; doi:10.1371/journal.pone.0211546)
Supplement: S2 Table — (DOCX) [file pone.0211546.s003.docx]

Table S2: Distance to SB of each factor within corresponding BN.

|  | All | Young female | Young male | Adult female | Adult male | Middle aged  female | Middle aged  male | Older adults  female | Older adults  male |
| --- | --- | --- | --- | --- | --- | --- | --- | --- | --- |
| Distance of nodes to SB |  |  |  |  |  |  |  |  |  |
| Sex | *2* |  |  |  |  |  |  |  |  |
| Age | *2* |  |  |  |  |  |  |  |  |
| **Psychology and behaviour** |  |  |  |  |  |  |  |  |  |
| Life satisfaction | *2* | NA | *2* | 3 | *2* | 3 | 3 | *2* | 3 |
| Internet use | *2* | 3 | *2* | *2* | 3 | *2* | 3 | 4 | 5 |
| **Institutional and home settings** | |  |  |  |  |  |  |  |  |
| Occupational level | ***1*** | ***1*** | ***1*** | ***1*** | ***1*** | ***1*** | ***1*** | 6 | NA |
| Educational level | *2* | *2* | NA | 3 | *2* | 3 | *2* | 3 | 8 |
| Social class | *2* | NA | *2* | *2* | ***1*** | *2* | *2* | 4 | 7 |
| Financial burden | ***1*** | NA | 3 | 3 | *2* | 4 | 3 | 3 | 4 |
| Wealth | ***1*** | NA | NA | *2* | 3 | 3 | 3 | 6 | 3 |
| Computer | 3 | 4 | 4 | 3 | 4 | 3 | 4 | 5 | 6 |
| Internet | *2* | 4 | 3 | 3 | 3 | 3 | 3 | 5 | 6 |
| Social media penetration | *2* | NA | NA | 3 | 4 | 3 | 3 | 5 | 3 |
| **Physical health and wellbeing** | |  |  |  |  |  |  |  |  |
| Quality of healthcare | *2* | NA | 3 | 4 | 3 | 4 | 4 | 3 | 4 |
| Healthcare provision | *2* | NA | NA | *2* | 4 | *2* | *2* | 3 | *2* |
| Prevalence of chronic diseases | 3 | NA | NA | 4 | 5 | 4 | 4 | 5 | 4 |
| **Built and natural environment** | |  |  |  |  |  |  |  |  |
| Urbanity | ***1*** | NA | NA | ***1*** | 3 | ***1*** | ***1*** | *2* | ***1*** |
| Availability of facilities | *2* | NA | NA | *2* | 4 | *2* | *2* | ***1*** | *2* |
| Support from municipality | *2* | NA | NA | 3 | 5 | 3 | 3 | *2* | ***1*** |
| Region | ***1*** | NA | NA | 3 | 4 | 3 | 3 | 4 | 3 |
| Precipitation | *2* | NA | NA | 3 | 4 | 3 | 3 | 4 | 3 |
| Temperature | *2* | NA | NA | 3 | 5 | 3 | 3 | 4 | 3 |
| **Social and cultural context** |  |  |  |  |  |  |  |  |  |
| Household size | *2* | 3 | 4 | ***1*** | NA | 5 | 4 | 6 | 8 |
| Having a Partner | *2* | *2* | *2* | *2* | NA | *2* | 3 | 7 | 3 |
| No. of Children | *2* | *2* | 3 | 3 | NA | 4 | 4 | 5 | 7 |
| Club membership | *2* | NA | NA | 2 | 2 | 3 | 3 | 6 | 8 |
| EU-migrant | *2* | NA | NA | 5 | 4 | NA | 4 | 6 | NA |
| **Politics and economics** |  |  |  |  |  |  |  |  |  |
| Car ownership | *2* | NA | NA | *2* | *2* | *2* | *2* | 6 | *2* |
| SB guidelines | *2* | NA | NA | 5 | 4 | 5 | 4 | 6 | 4 |
| PA guidelines | 3 | NA | NA | 4 | 5 | 3 | 4 | 5 | 4 |
| Transport policy | *2* | NA | NA | 4 | 5 | 4 | 4 | 6 | 3 |
| GDP | ***1*** | NA | NA | 4 | 3 | 4 | 3 | 5 | 3 |
