# Supplementary figures and images for: Factors influencing sedentary behaviour: A system based analysis using Bayesian networks within DEDIPAC
Source: PLoS One. 2019 Jan 30;14(1):e0211546. doi: 10.1371/journal.pone.0211546 (PMC6353197; doi:10.1371/journal.pone.0211546)

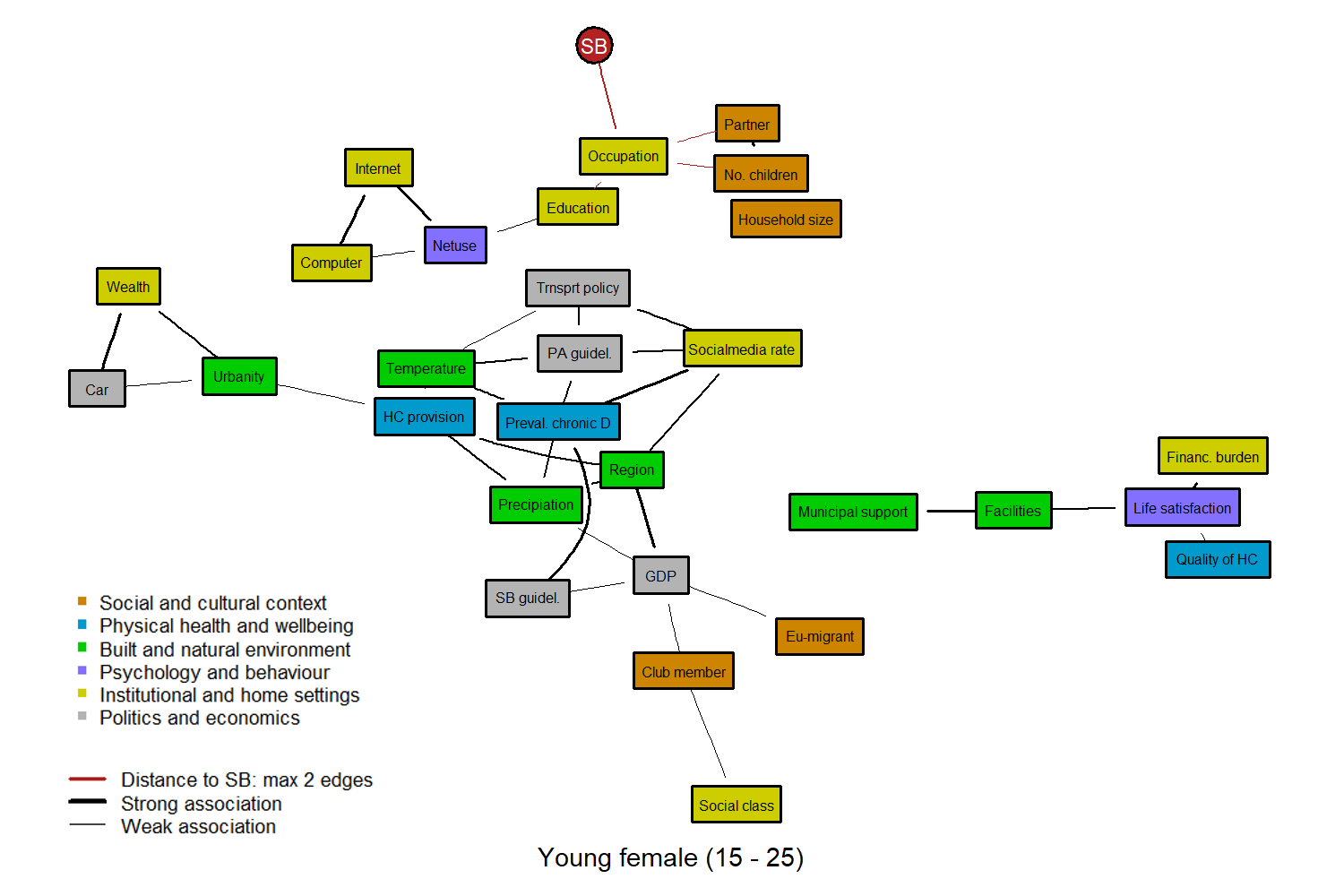

Supplement: S1 Fig — (TIFF) [file pone.0211546.s004.tiff]

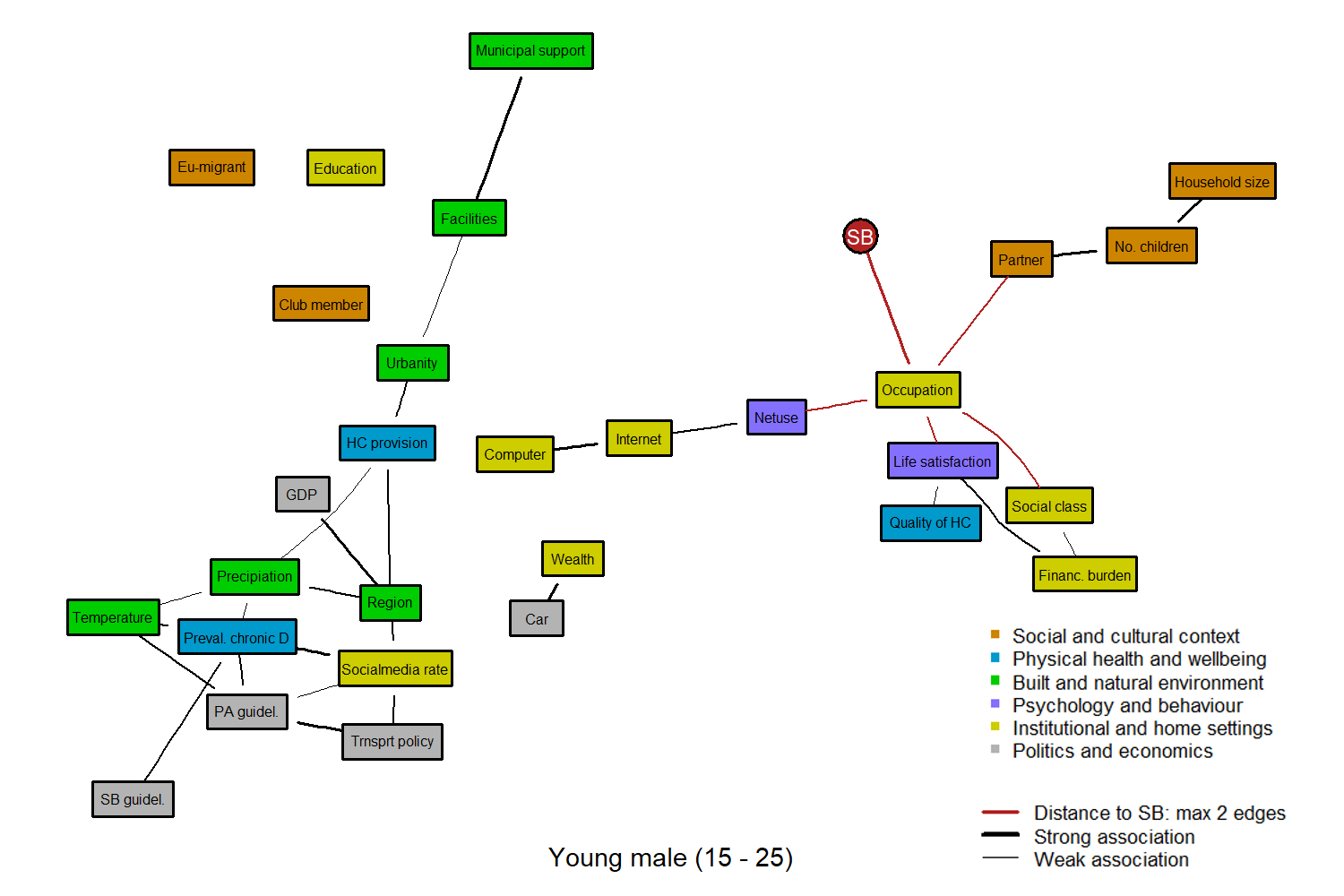

Supplement: S2 Fig — (TIFF) [file pone.0211546.s005.tiff]

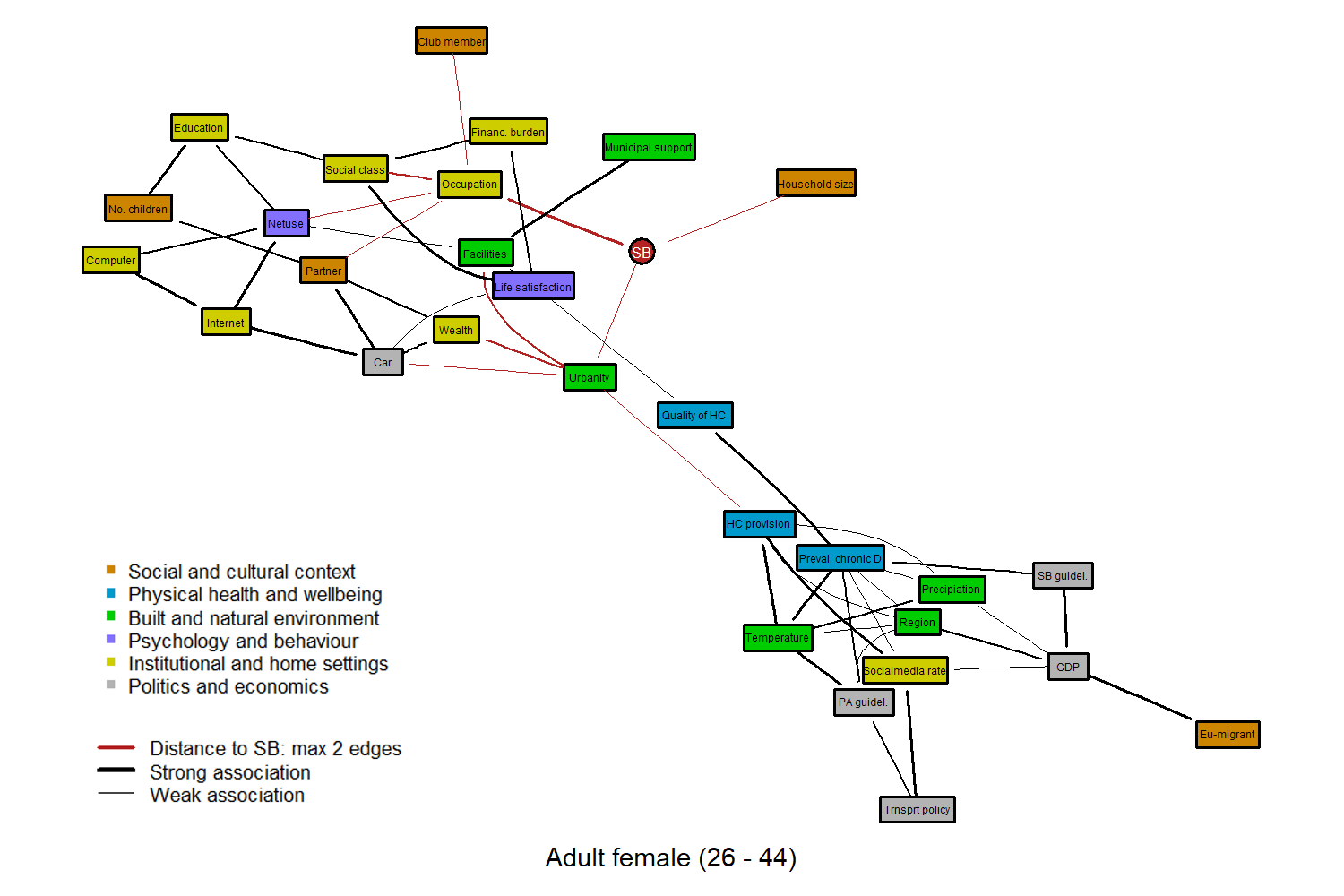

Supplement: S3 Fig — (TIFF) [file pone.0211546.s006.tiff]

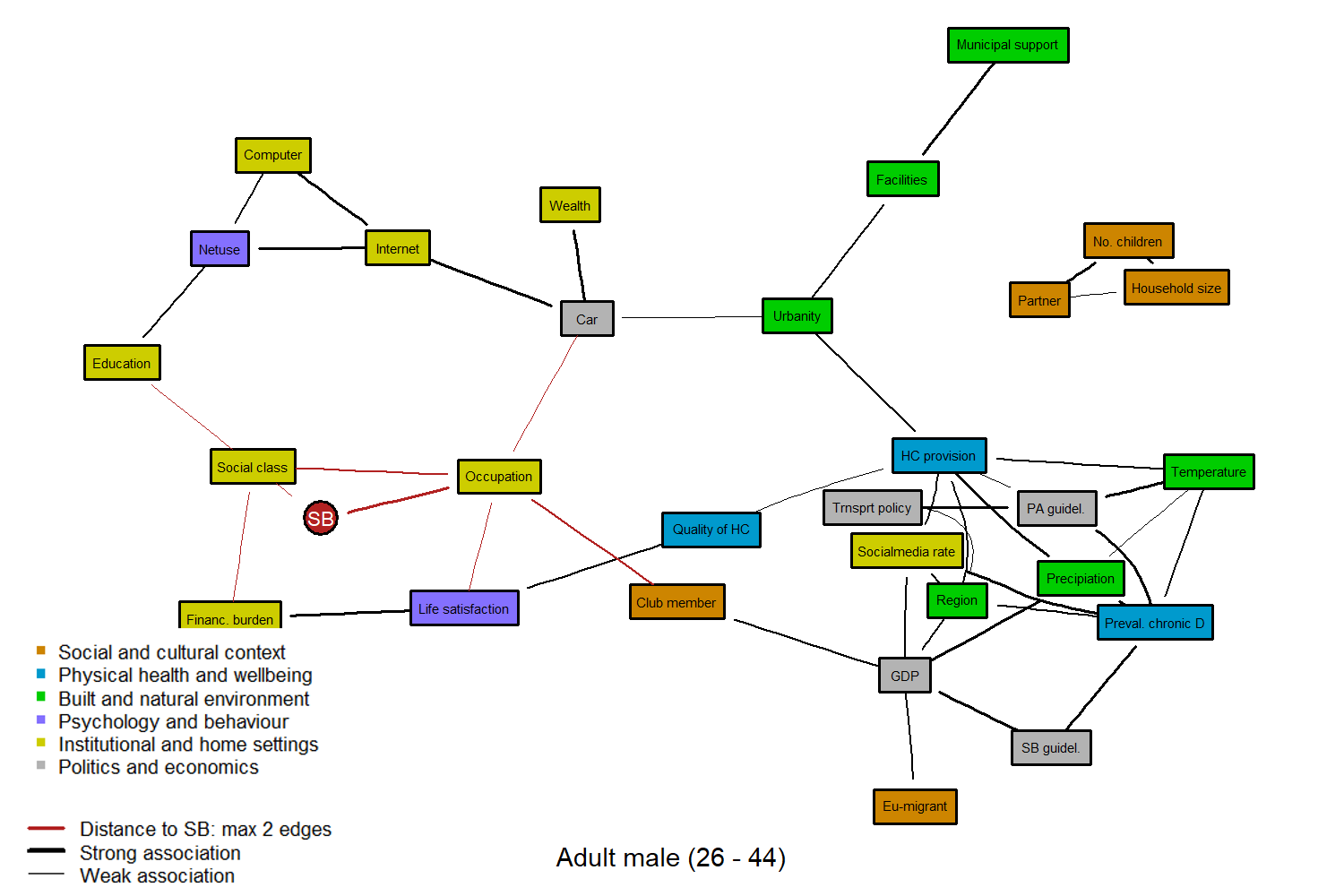

Supplement: S4 Fig — (TIFF) [file pone.0211546.s007.tiff]

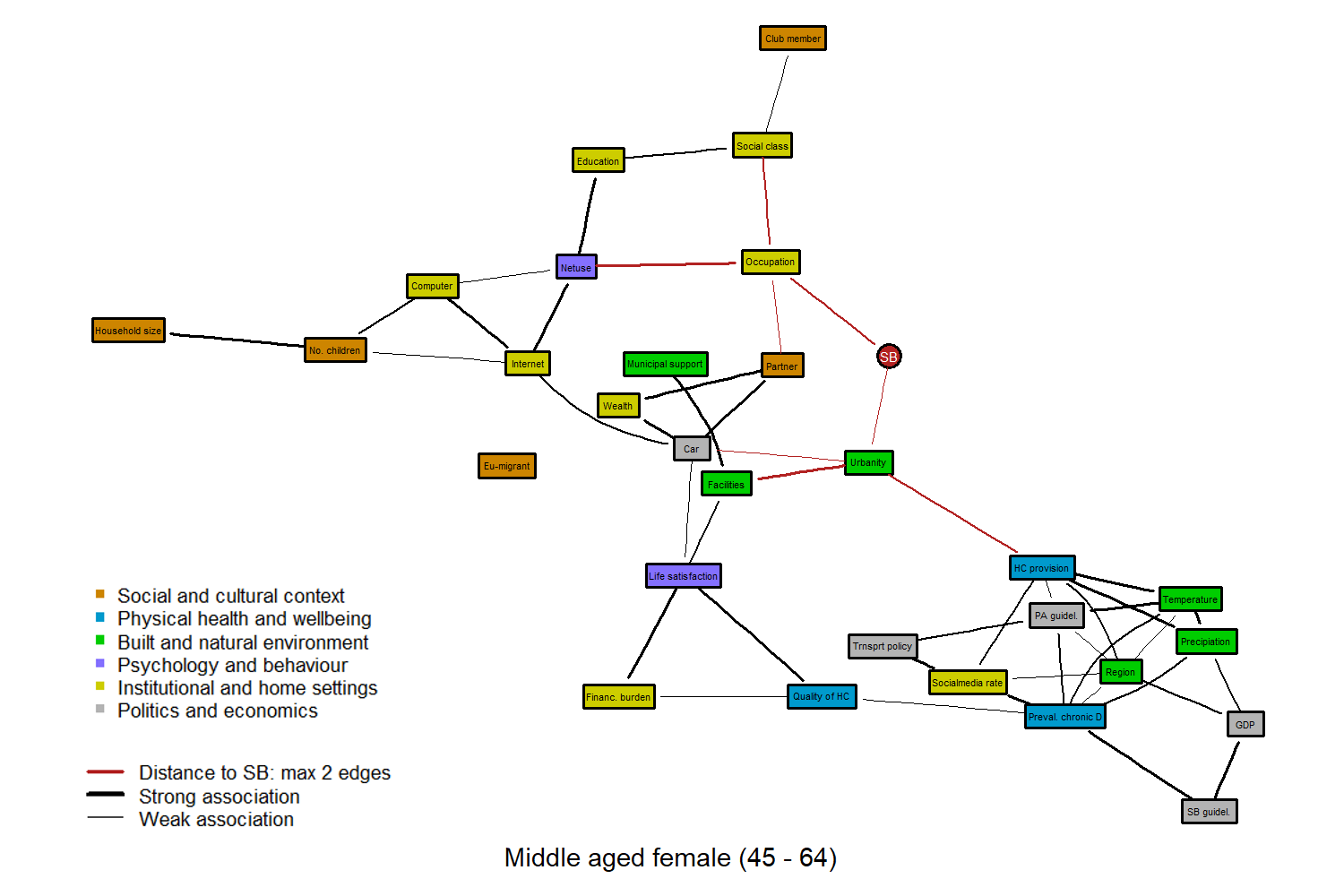

Supplement: S5 Fig — (TIFF) [file pone.0211546.s008.tiff]

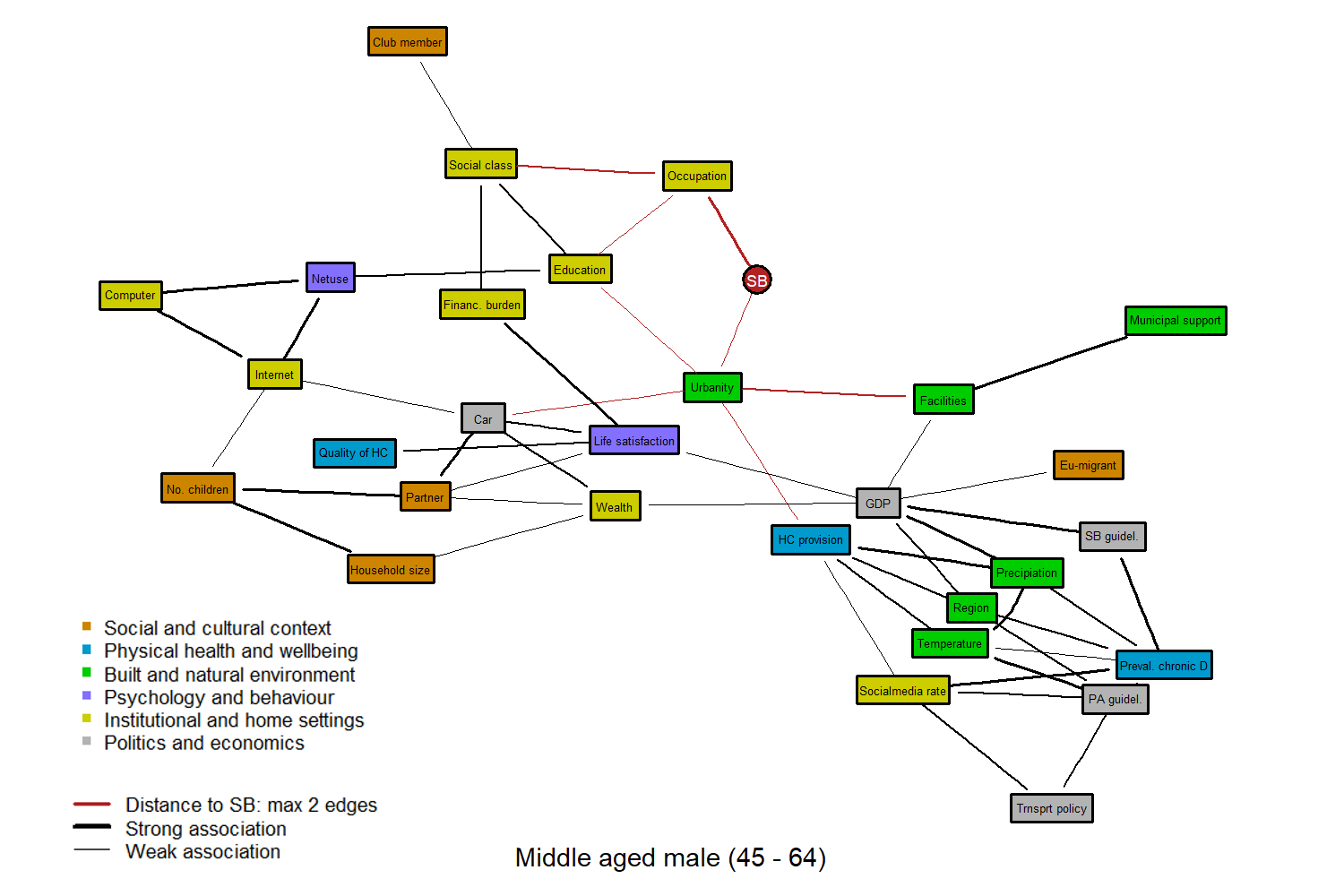

Supplement: S6 Fig — (TIFF) [file pone.0211546.s009.tiff]

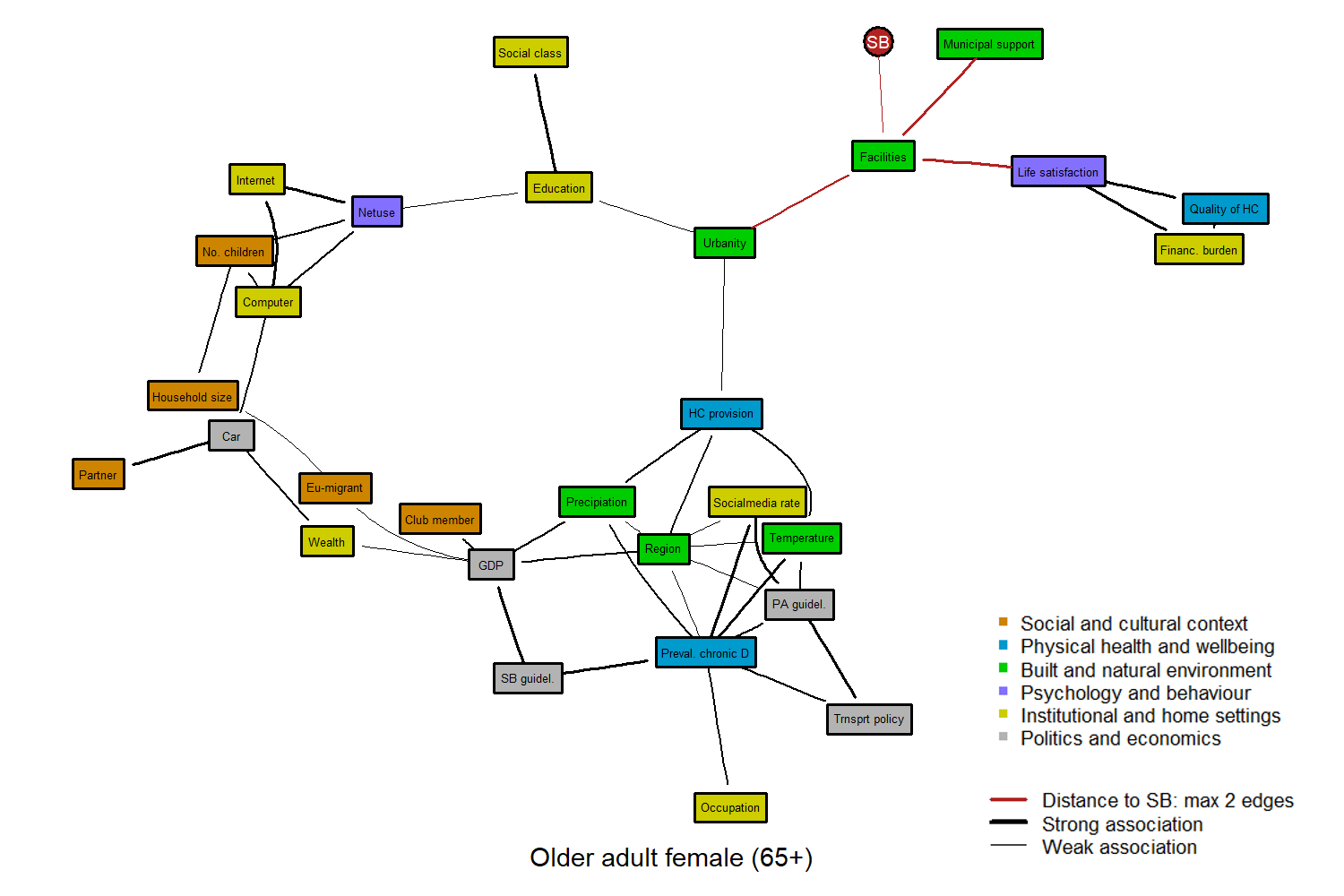

Supplement: S7 Fig — (TIFF) [file pone.0211546.s010.tiff]

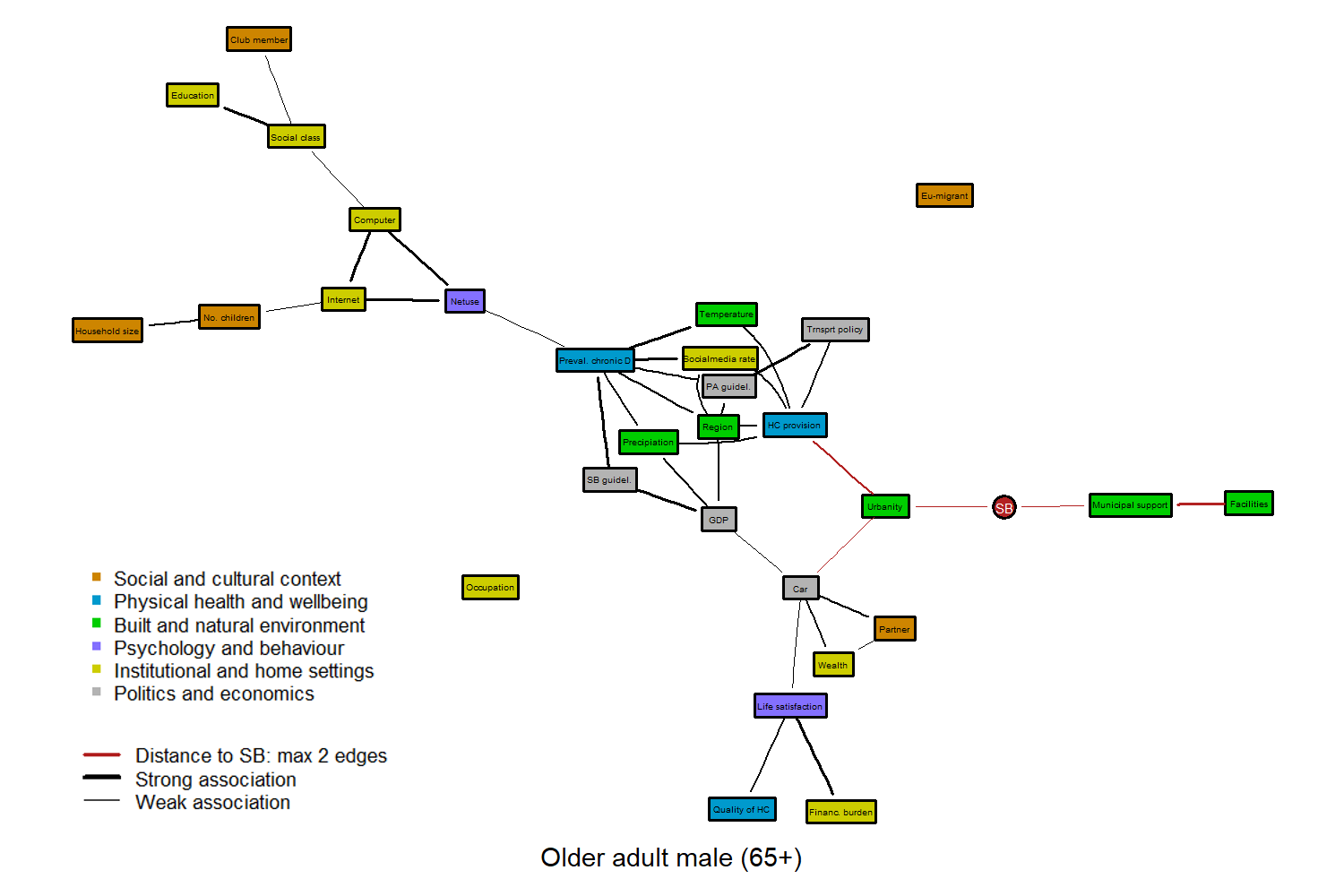

Supplement: S8 Fig — (TIFF) [file pone.0211546.s011.tiff]
